# The 1.3 Å resolution structure of the truncated group Ia type IV pilin from *Pseudomonas aeruginosa* strain P1

**DOI:** 10.1107/S205979832401132X

**Published:** 2024-11-28

**Authors:** Nicholas Bragagnolo, Gerald F. Audette

**Affiliations:** a Department of Chemistry York University 4700 Keele Street Toronto OntarioM3J 1P3 Canada; University of Melbourne, Australia

**Keywords:** type IV pilins, *Pseudomonas aeruginosa*, AI-generated models, SEC-MALS, *ProSMART* structural comparisons

## Abstract

The type IV pilus is a diverse molecular machine capable of conferring a variety of functions and is produced by a wide range of bacterial species. The ability of the pilus to perform host-cell adherence makes it a viable target for the development of vaccines against infection by human pathogens such as *Pseudomonas aeruginosa*. Here, the 1.3 Å resolution crystal structure of the N-terminally truncated type IV pilin from *P. aeruginosa* strain P1 (ΔP1) is reported, the first structure of its phylogenetically linked group (group I) to be discussed in the literature. The structure was solved from X-ray diffraction data that were collected 20 years ago with a molecular-replacement search model generated using *AlphaFold*; the effectiveness of other search models was analyzed. Examination of the high-resolution ΔP1 structure revealed a solvent network that aids in maintaining the fold of the protein. On comparing the sequence and structure of P1 with a variety of type IV pilins, it was observed that there are cases of higher structural similarities between the phylogenetic groups of *P. aeruginosa* than there are between the same phylogenetic group, indicating that a structural grouping of pilins may be necessary in developing antivirulence drugs and vaccines. These analyses also identified the α–β loop as the most structurally diverse domain of the pilins, which could allow it to serve a role in pilus recognition. Studies of ΔP1 *in vitro* polymerization demonstrate that the optimal hydrophobic catalyst for the oligomerization of the pilus from strain K122 is not conducive for pilus formation of ΔP1; a model of a three-start helical assembly using the ΔP1 structure indicates that the α–β loop and the D-loop prevent *in vitro* polymerization.

## Abstract 2

The structure of a type IV pilin from a human-pathogenic strain of *P. aeruginosa* was solved to 1.3 Å resolution using molecular replacement with an *AlphaFold*-generated search model. It is the first structure from this group of pilins to be discussed and reveals the structural differences that result in functional diversity between closely related type IV pili.

## 1. Introduction

### 1.1. Importance of the type IV pilus from *Pseudomonas aeruginosa*

*Pseudomonas aeruginosa* is the most common pathogen of humans and warm-blooded mammals from the group of Proteobacteria known as the nonfermenting Gram-negative bacteria (NFGNB; Behzadi *et al.*, 2021 ▸; Haenni *et al.*, 2017 ▸). As *P. aeruginosa* is a waterborne, obligative aerobic, non­fastidious bacterial species that can survive over a wide temperature range of 4–40°C, it often colonizes the respiratory system of mammals (Curran *et al.*, 2018 ▸; Williams *et al.*, 2010 ▸; Behzadi *et al.*, 2021 ▸). It is a well known opportunistic pathogen of the lungs of immunocompromised patients such as those with healthcare-associated pneumonia, chronic obstructive pulmonary disease (COPD) or cystic fibrosis (CF) (Curran *et al.*, 2018 ▸; Williams *et al.*, 2010 ▸; Jurado-Martín *et al.*, 2021 ▸). *P. aeruginosa* infections are associated with high morbidity and mortality, and with the emergence of many novel multidrug-resistant (MDR) strains found in nosocomial settings this bacterium has been included in the ‘critical’ category of the World Health Organization’s priority list of bacterial pathogens for which research and development of new antibiotics are urgently needed (Jurado-Martín *et al.*, 2021 ▸). The prevalence of novel MDR *P. aeruginosa* strains in the healthcare setting has been further exacerbated by the COVID-19 pandemic, which saw many uncharacterized co-infections of *P. aeruginosa*, likely due to the overprescription of broad-spectrum antibiotics and the use of ventilators, which are common fomites for this species (Bongiovanni & Barda, 2023 ▸; Ng *et al.*, 2023 ▸; Ramírez-Estrada *et al.*, 2016 ▸).

There is an expansive arsenal of mechanisms of secretion that *P. aeruginosa* uses to adapt to various environments; chief among them are type IV pili (T4P), which are responsible for a variety of bacterial functions (Jurado-Martín *et al.*, 2021 ▸). They confer the main method of attachment of *P. aeruginosa* by binding to a receptor on host mucosal cells for pathogenicity, provide twitching motility across surfaces and aid in microcolony and biofilm formation, cell signaling, DNA uptake and regulating virulence-gene expression (Lento *et al.*, 2016 ▸; Van Schaik *et al.*, 2005 ▸; Persat *et al.*, 2015 ▸; Klausen *et al.*, 2003 ▸). The T4P is a filamentous polymer made of type IV pilins, the individual protein monomer (PilA), that is polymerized by several chaperones and minor pilins to form fibers that can extend micrometres in length with an outer diameter of ∼5–8 nm (Wang *et al.*, 2017 ▸; Lento *et al.*, 2016 ▸; McCallum *et al.*, 2019 ▸). Pilin monomers are assembled into a pilus and disassembled by a membrane-spanning complex known as the type IV pilus machinery that is evolutionarily and structurally related to the type II secretion system. There are two main T4P subtypes based on sequence homology in the pilin protein: type IV a (T4aP) and type IV b (T4bP) pili. The T4aP are present in a broad species range including *P. aeruginosa*, whereas T4bP are a heterogeneous group common in enteric species such as *Escherichia coli* and *Vibrio cholerae* (Roux *et al.*, 2012 ▸; McCallum *et al.*, 2019 ▸). Disruption of the T4P has been shown to greatly reduce the virulence of Gram-negative bacterial pathogens, such that type IV pilin proteins have been considered to be promising targets for the development of antivirulence drug targets and vaccine/antibody therapy candidates (Giltner *et al.*, 2012 ▸; Lento *et al.*, 2016 ▸; Campbell *et al.*, 1997 ▸; Hertle *et al.*, 2001 ▸; Horzempa *et al.*, 2008 ▸). Another relevance to the biomedical field is the use of T4Ps in the formation of self-assembling protein nanotubes (PNTs) that have significant potential as targeted drug-delivery vehicles due to their low immunogenicity and the ability to design the receptor-binding domain of the pilin to target various surfaces (Audette *et al.*, 2019 ▸; Petrov *et al.*, 2013 ▸; Giltner *et al.*, 2006 ▸).

### 1.2. Structural aspects of the T4P pilin protein

The general architecture of the type IV pilin protein is an extended N-terminal α-helix connected by an α–β loop to a four- to seven-stranded β-sheet, termed the head domain, which packs onto one half of the α-helix (Hazes *et al.*, 2000 ▸; Giltner *et al.*, 2012 ▸). The N-terminal helix is split into two subdomains; the α1-N region is highly hydrophobic and protrudes from the globular C-terminal domain, while the α1-C region embedded in the C-terminal globular head domain is amphipathic and packs against the head domain. The α1-N region is multifunctional; it serves as a transmembrane anchor to hold pilin subunits in the cytoplasmic lipid bilayer prior to assembly, it is the protein-interaction domain for pilin–pilin interactions to form the central core in the assembled pilus polymer and it has been predicted to serve as a regulatory domain for transduction of the pilus-attachment signal to induce virulence-gene expression (Giltner *et al.*, 2012 ▸; Persat *et al.*, 2015 ▸). The N-terminal helix is highly conserved in *P. aeruginosa* pilins; sequence variations in the α–β loop and in the hypervariable loops connecting the β-sheets in the head domain provide structural diversity between pilin proteins from different bacteria. While there are variations in the protein sequence, the β-sheet of type IV pilin proteins is a conserved structural motif composed of a β-meander with 3–4 β-strands having nearest-neighbor connectivity. The C-terminal globular head domain faces the exterior of the assembled pilus, and in *P. aeruginosa* pilin proteins it features a conserved disulfide-bonded loop region that is responsible for cellular and surface adhesion termed the D-region.

Five different groups of T4aP have been identified in *P. aeruginosa*, classified by sequence length, the size of the D-region and the identity of the pilin accessory protein encoded downstream of the pilin (Kus *et al.*, 2004 ▸). Group II pilins are the simplest as they lack accessory genes between *pilA_II_* and the downstream *tRNA^Thr^* gene, whereas group I pilins are slightly larger proteins and have an ORF between the two accessory genes that is responsible for glycosylation of the C-terminal serine termed *pilO* or *tfpO*. Group I is also split into two subgroups (1a and 1b) based on the sequence homology of the *pilA_I_* and *tfpO* genes. *P. aeruginosa* strains PAO1, PAK, CD, KB7 and K122-4 express group II pilins; however, strains P1 and 1244 express group Ia pilins and strain SBI-N expresses a group Ib pilin. Group III pilins such as those found in PA14 are longer, at 173 residues, and have an ORF immediately downstream of the *pilA_III_* gene called *tfpY* that is responsible for enhanced twitching motility and an increased number of surface pili (Asikyan *et al.*, 2008 ▸). Group IV pilins share higher sequence homology to the minor pilin PilE from *Neisseria meningitidis* than to the *P. aeruginosa* pilin proteins from other groups, and two accessory proteins from genes *tfpW* and *tfpX* are encoded immediately downstream of *pilA_IV_*, with functions of a glycosyltransferase/oligosaccharyltransferase and an unknown function similar to that of TfpY, respectively (Kus *et al.*, 2008 ▸; Villela *et al.*, 2020 ▸; Asikyan *et al.*, 2008 ▸). Group V pilins such as the pilin from *P. aeruginosa* strain 110594 are a single residue shorter than group III pilins, and they have a single accessory protein encoded by *tfyZ* downstream of the pilin gene, which is homologous to TfpY and TfpX in sequence and function (Asikyan *et al.*, 2008 ▸; Kus *et al.*, 2004 ▸; Giltner *et al.*, 2012 ▸; Nguyen *et al.*, 2010 ▸).

### 1.3. Solving the structure of the P1 pilus

In 1984, sputum samples of CF patients with *P. aeruginosa* infections were cultured from the University of Minnesota Hospital, Minneapolis, and a strain was serotyped P1 for a study on the effect of nonopsonic phagocytosis on different bacterial strains (Speert *et al.*, 1984 ▸). Pasloske *et al.* (1988 ▸) reported the cloning and sequencing of the pilin genes from strains P1 and K122-4, identifying their sequence features as differing from the model *P. aeruginosa* pilins, the PAK, PAO and CD pilins (Pasloske *et al.*, 1988 ▸). The sequence of P1 was determined to be nearly identical, save for one residue (K92Q), to that of strain 1244, another clinical isolate (Castric *et al.*, 1989 ▸; Pasloske *et al.*, 1988 ▸). In 2004, the crystal structure of the truncated K122-4 (ΔK122) pilin [PilA(Δ1–28)] was solved; crystals of the N-terminally truncated P1 (ΔP1) pilin [PilA(Δ1–35)] were obtained and diffracted in a similar manner, but the structure could not be solved due to a lack of anomalous signal in heavy-atom-soaked crystals, and the structure of the ΔP1 pilin could not be solved using molecular replacement (MR) due to insufficient similarity to proteins with solved structures (Audette, Irvin *et al.*, 2004 ▸; Audette *et al.*, 2003 ▸).

In 2021, when *AlphaFold* became accessible, a predictive model of ΔP1 was generated; many have demonstrated the ability of the algorithm to generate protein models suitable for use in MR (Jumper *et al.*, 2021 ▸; Oeffner *et al.*, 2022 ▸; McCoy *et al.*, 2022 ▸; Barbarin-Bocahu & Graille, 2022 ▸). Prior to this, a reliable MR search model for the ΔP1 pilin was not readily available, although the structure of the truncated 1244 (Δ1244) pilin had been submitted to the PDB (entry 6bbk; Y. Shen, Y. Nguyen, A. Guarne & L. L. Burrows, unpublished work). In this report, we demonstrate how the phase problem was solved using MR with a processed search model generated by the *ColabFold* algorithm (Mirdita *et al.*, 2022 ▸). The ΔP1 pilin structure was refined to 1.30 Å resolution, allowing a resolved view of side-chain positioning and important solvent contacts. An analysis of *Phaser* results using different MR models demonstrates that use of a processed *AlphaFold* model enhances the quality of the initial maps compared with using a nearly identical structure from the Δ1244 pilin. Size-exclusion chromatography linked to multi-angle light scattering (SEC-MALS) on ΔP1 after attempting *in vitro* oligomerization provides evidence of differing behaviors between ΔP1 and ΔK122. Analyses in *PISA* (*Protein Interfaces, Surfaces and Assemblies*), *ProSMART* (*Procrustes Structural Matching Alignment and Restraints Tool*) and *Clustal Omega* compared pilin structures from different pilin groups (Krissinel & Henrick, 2007 ▸; Nicholls *et al.*, 2014 ▸; Thompson *et al.*, 1994 ▸). Our results indicate that structural homologies may exist between pilins from different groupings, thus displaying the need for further structural analysis of *P. aeruginosa* pilins to obtain a more nuanced structural grouping and to aid in antivirulence drug development.

## 2. Materials and methods

### 2.1. Expression and purification of ΔP1

Cloning, expression and purification of the ΔP1 pilin were performed in a similar fashion to that previously reported for ΔK122 (Audette *et al.*, 2003 ▸). Briefly, the P1 pilin gene was excised from a pRLD plasmid containing *pilA_P1_* by restriction digestion using EcoRI and HindIII. DNA was isolated from a 1.2% agarose gel using standard procedures and was ligated in-frame into the pMAL-p2 expression vector (NEB). ΔP1 [*pilA(Δ1–28)*] was expressed periplasmically in *E. coli* strain ER2507 as a maltose-binding protein (MBP) fusion protein and was purified using an amylose column. The purified MBP-ΔP1 fusion protein was trypsinized to release ΔP1 pilin from MBP. The monomeric ΔP1 pilin contains four N-terminal residues (ISEF) from the expression construct followed by residues 28–148 of the P1 pilin (this numbering scheme excludes the six-residue cleaved signal sequence, MKAQKG). Monomeric ΔP1 was then purified by cation-exchange chromatography and quantified using UV_280 nm_ spectroscopy and the known molar absorptivity coefficient of 0.694 ml mg^−1^ cm^−1^.

### 2.2. Crystallization and structural determination of ΔP1

All crystallization experiments were performed using the hanging-drop vapor-diffusion method at 295 K. Initial crystals of the ΔP1 pilin were grown in 1–2 days from 2 µl drops containing equal volumes of protein (20 mg ml^−1^ in 10 m*M* Tris pH 7.4, 100 m*M* sodium chloride) and reservoir solution [35%(*w*/*v*) PEG 4000, 100 m*M* sodium cacodylate pH 6.0, 100 m*M* monobasic potassium phosphate]. These initial crystals were used as a microseed stock for further crystallization experiments. Crystals suitable for X-ray diffraction analysis (typical dimensions of ∼180 × 60 × 30 µm) were grown from 3 µl drops containing equal volumes of protein, reservoir [30%(*w*/*v*) PEG 4000, 100 m*M* sodium cacodylate pH 6.0, 100 m*M* monobasic potassium phosphate] and microseed solutions. Crystals were mounted in cryoloops (Hampton Research) and flash-cooled by direct immersion into liquid nitrogen prior to X-ray diffraction analysis.

X-ray diffraction data were collected from a single crystal of ΔP1 (dimensions of ∼180 × 60 × 30 µm) on beamline 8.3.1 at the Advanced Light Source (ALS) in 2003 using an ADSC Quantum 315 CCD detector. Diffraction data were collected at 100 K in two passes. The first, lower resolution data set was collected with the following experimental parameters: a 300 mm crystal-to-detector distance and λ = 1.0236 Å, 10 s per exposure and an angular range covering 0–90° in a total of 90 frames with 1.0° per image. The second, high-resolution data set was collected with the following experimental parameters: a 150 mm crystal-to-detector distance and λ = 1.0332 Å, 30 s per exposure and an angular range covering 0–146° in a total of 292 frames with 0.5° per image. The low- and high-resolution passes were indexed using *MOSFLM* (Leslie, 2006 ▸), following which the indexed reflections were merged and scaled with *SCALA* from the *CCP*4 suite to 1.20 Å resolution. At the time, structure solution of the ΔP1 pilin was unfortunately unsuccessful (either by MR or anomalous signal phasing). The images were archived, with only the indexed data files (one each for the low- and high-resolution pass) remaining available. We recently revisited the merging and scaling of the *MOSFLM* indexed data employing *AIMLESS* from the *CCP*4 suite (Evans & Murshudov, 2013 ▸), identifying a more appropriate resolution cutoff as 1.3 Å based on CC_1/2_ and other more recently deployed metrics, and selecting 3% of reflections for *R*_free_ comparison in monitoring the *R*_work_ value during refinement. ΔP1 crystallized with a single monomer in the asymmetric unit and 30.72% solvent content; data-collection statistics for ΔP1 are shown in Table 1 ▸.

The structure was solved using molecular replacement by *Phaser* (McCoy *et al.*, 2007 ▸) via the *CCP*4*i* GUI (Potterton *et al.*, 2003 ▸) using an *AlphaFoldab initio* model generated using *ColabFold* (version 1.5.2) that was processed using the ‘process predicted model’ function in *CCP*4*i*2, which generates a model separated into discrete regions based on the predicted local distance difference test (pLDDT) score of the model (Mirdita *et al.*, 2022 ▸; Agirre *et al.*, 2023 ▸). The MR result was then input into *ARP*/*wARP*, resulting in a solution with *R* and *R*_free_ values of 0.1848 and 0.256, respectively, due to dummy glycine residues in the place of the N-terminal Ile25–Thr67 and the C-terminal Ser147. The model was then examined in *Coot* (Emsley & Cowtan, 2004 ▸; Emsley *et al.*, 2010 ▸), side chains were manually assigned and a number of residues showed occupancy in multiple conformations [Thr34 (0.5, 0.5), Thr45 (0.5, 0.5), Ser118 (0.5, 0.5), Lys120 (0.5, 0.5), Cys127 (0.8, 0.2), Lys128 (0.6, 0.4) and Pro146 (0.5, 0.5)]. Solvent atoms were added with *ARP*/*wARP* and confirmed by visual inspection; solvent density corresponding to a cacodylic acid (dimethylarsinic acid) molecule was observed and modeled accordingly. The model was processed in several rounds of remodeling and refinement performed in *Coot* and *REFMAC*5, respectively (Langer *et al.*, 2008 ▸; Murshudov *et al.*, 2011 ▸; Morris *et al.*, 2003 ▸; Emsley *et al.*, 2010 ▸). Restrained individual anisotropic *B* values were refined in the final cycle prior to validation using *SFCHECK*, *PROCHECK* and *RAMPAGE* (Murshudov *et al.*, 2011 ▸; Lovell *et al.*, 2003 ▸; Laskowski *et al.*, 1993 ▸; Vaguine *et al.*, 1999 ▸). The validity of the anisotropic *B*-value refinement at this resolution was supported by the *R*_free_ statistic, which decreased from 0.1987 to 0.1738. The final model contains 124 residues, a single cacodylic acid molecule and 216 waters; refinement statistics are summarized in Table 1 ▸ and the final model and structure-factor amplitudes were submitted to the RCSB Protein Data Bank with accession code 8v7p.

### 2.3. Sequence and structure comparisons

*PISA* (*Protein Interfaces, Surfaces and Assemblies*) analysis was performed on the ΔP1 structure using *PDBePISA* version 1.52 with the processing mode set to auto (Krissinel & Henrick, 2007 ▸). Interface results were used to search for other proteins with similar interfaces, and monomers and assemblies were analyzed to view potential quaternary structures that can form based on the ΔP1 structure. Model *P. aeruginosa* pilin proteins with solved structures and structures with similar interfaces as ΔP1 from *PISA* results were compared using *ProSMART* (*Procrustes Structural Matching Alignment and Restraints Tool*; Nicholls *et al.*, 2014 ▸). *ProSMART ALIGN* was used from the *CCP*4*i* interface with default settings to compare the structures in a conformation-independent manner. Multiple sequence alignment of the pilins was performed using *Clustal Omega* for comparison with structural alignment values; the results were visualized in *JalviewJS* version 2.12 (Waterhouse *et al.*, 2009 ▸; Sievers & Higgins, 2018 ▸).

### 2.4. SEC-MALS

The ΔP1 pilin was purified as described above and was concentrated to 23 mg ml^−1^ using a 5 kDa molecular-weight cutoff (MWCO) centrifugal concentrator (Millipore) at 3500*g* and 4°C. The protein sample was injected at a concentration of 20 mg ml^−1^ and a volume of 100 µl onto a Yarra s3000 size-exclusion chromatography (SEC) column (separation range 5–700 kDa; Phenomenex) using an ÄKTApurifier 10S FPLC system (Cytiva) connected inline to a Dawn Heleos II multi-angle light scattering (MALS) system and an Optilab T-rEX direct refractive-index (dRI) detector (Wyatt Technology). The running buffer used was 20 m*M* Tris, 150 m*M* NaCl pH 7.4 and was loaded at a flow rate of 0.5 ml min^−1^.

Petrov *et al.* (2013 ▸) characterized the oligomerization of ΔK122 into protein nanotubes (PNT) using a variety of hydrophobic trigger molecules and determined the optimal catalyst to be 2-methyl-2,4-pentanediol (MPD). A similar procedure was followed to oligomerize ΔP1 into PNT through the addition of trigger solution (10 m*M* Tris, 300 m*M* NaCl, 1 m*M* EDTA, 1 m*M* DTT, 1 *M* MPD pH 7.4) to 23 mg ml^−1^ ΔP1 in a 10:1(*v*:*v*) protein:hydrophobe ratio. The oligomerization reaction was incubated at room temperature with nutation for 96 h. SEC-MALS was performed in the same manner as described above; analysis of SEC-MALS data was performed using the *ASTRA* software package (version 6.0) and provided hydrodynamic radii (*R*_h_), molar mass, polydispersity and translational diffusion coefficient (*D*_t_) distributions as calculated from an autocorrelation function. Based on SEC-MALS data and previous experimental data showing a three-start helical assembly for the oligomerization of pilins, a model of the assembled P1 pilus was created using the truncated P1 monomeric structure (Petrov *et al.*, 2013 ▸; Craig *et al.*, 2006 ▸).

## 3. Results and discussion

### 3.1. Quality of the final model

The structure of the monoclinic crystal form of ΔP1 pilin was refined to 1.3 Å resolution, with a final *R*_work_ and *R*_free_ of 0.1306 and 0.1738, respectively (Table 1 ▸). In the final model, all residues except the N-terminal side chain (Ile25) were observed, and 216 water molecules and a cacodylic acid molecule could be modeled in the electron density. Ile25 and Thr66 had a poor fit to the electron density, with RSR *Z*-scores (Gore *et al.*, 2017 ▸) of 4.4% and 2.6%, respectively. The high RSR *Z*-score of Ile25 is common for the first residue of the protein chain as terminal residues typically have higher conformational disorder; also, Ile25 is a residue resulting from the N-terminal MBP tag and therefore is not relevant in discussing the native ΔP1 structure. The average *B* factors for the main chain and side chains are 10.02 and 13.21 Å^2^, respectively, and that for the solvent molecules is 27.21 Å^2^. Analysis of the stereochemical quality of the final model using *PROCHECK*, *SFCHECK* and *RAMPAGE* indicated that 97.5% of residues (120) are in the core or allowed regions of the Ramachandran plot, and only 2.5% of residues (Asp65, Thr73 and Lys104) are in less favored regions; however, their side-chain r.m.s.d. values were shown to be reasonable (0.0624, 0.1090 and 0.0638 Å, respectively) as the side chains were properly modeled into the density (Laskowski *et al.*, 1993 ▸; Vaguine *et al.*, 1999 ▸; Lovell *et al.*, 2003 ▸; Chen *et al.*, 2010 ▸). A single side-chain planarity outlier was seen in Arg35, and a bond-angle outlier was seen in Arg119; no other strained stereochemical conformations were observed.

### 3.2. Features of the ΔP1 structure

**Figure 1:**
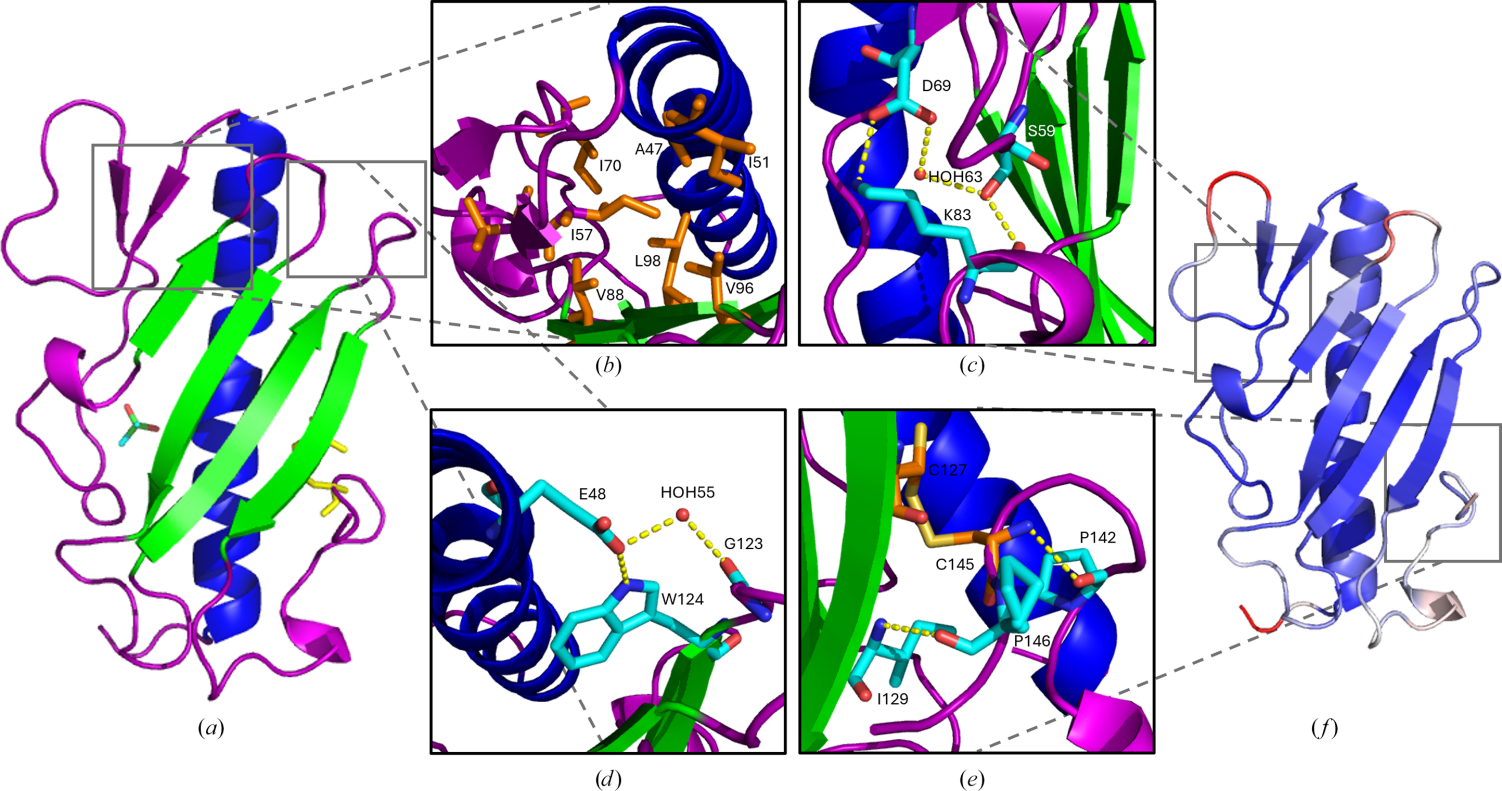
The structure of the ΔP1 pilin with important residue interactions highlighted (PDB entry 8v7p). (*a*) The ΔP1 pilin monomer; the N-terminal α-helix (α1-C) is in blue, the β-sheet is in green and the coil regions are in purple. The disulfide between Cys127 and Cys145 is shown in yellow and the cacodylic acid molecule is shown as sticks with atomistic coloring. (*b*) A portion of the hydrophobic interface seen between the α1-C helix, the β-meander and the α–β loop. Side chains of the hydrophobic residues (orange) are pointed towards the interior of the molecule to create a hydrophobic pocket. At 1.3 Å resolution numerous solvent contacts were seen in the ΔP1 structure, including the hydrogen-bonding network (dotted yellow lines) containing a water molecule (in red) seen in (*c*) which aids in maintaining the conformation of the α–β loop. (*d*) A view of the bend in the α1-C helix and the hydrogen bonding between the conserved Trp125 and Glu48, as well as the backbone of Gly123 and water 55, which stabilizes the kinked α-helix. (*e*) The receptor-binding D-loop; residues making hydrogen bonds supporting the disulfide bond. Cys127 is depicted with its reduced conformer and Pro146 is shown with both *endo* and *exo* conformers. (*f*) ΔP1 is colored via *B* factor, with low to high *B* factors set from blue to red (5–22 Å^2^). The highest *B* factors are in the α–β loop and hypervariable loop regions on the side opposite to the RBD, with some dynamics seen in the RBD. All images were generated using *PyMOL* version 2.5.0 (Schrödinger).

The ΔP1 crystal structure exhibits the characteristic type IVa pilin fold, with the truncated N-terminal α-helix packed onto the head domain consisting of a four-stranded antiparallel β-sheet (Fig. 1 ▸*a*). The α–β loop immediately follows the amphipathic α1-C helix, and the receptor-binding D-region with the conserved disulfide bond to the last strand of the β-meander is present. While these features are common, minor differences in T4 pilin structures result in the observed functional diversity of the *P. aeruginosa* pili; as no other T4a group I pilin structures have been described in the literature we will detail the side-chain interactions that result in the observed conformations of the α-helix, the β-sheet, the D-region and the connecting loop regions.

The α1-C helix has some interesting side-chain interactions that may be unique despite the region having high sequence conservation in other T4 pilin proteins. Some residues on the helix contribute to stabilizing the conformation of proximal hypervariable loop regions, such as Gln32, which has two conformers, one which uses the carboxyl group of the side chain to hydrogen-bond to the backbone N atom of Gly107 (2.99 Å), part of the β_2_–β_3_ loop. Glu39 hydrogen-bonds strongly to Ser75 (2.48 Å), which connects the α–β loop to the helix. Thr34 also has two conformers, one of which hydrogen-bonds to the backbone carbonyl groups of Arg30 and Thr31 (3.10 and 3.15 Å, respectively), presumably stabilizing the conformation of the helix. There is a shallow bend in the helix starting at Ser41 which offsets the hydrogen bonding of the main chain (Section S1). Another important residue to note in the α1-C region is Arg35, the only planarity outlier. Using N^η1^ of the side chain, Arg35 strongly hydrogen-bonds to Glu27 (2.40 Å), and N^η2^ is connected to the cacodylic acid through hydrogen bonding to water 8 (2.93 Å, *B* factor of 17.52 Å^2^), Glu39 (2.69 Å) and water 17 (2.75 Å, *B* factor of 20.59 Å^2^). The uncommon planarity of the Arg35 side chain may be due to these interactions and its high *B* factor (18.7 Å^2^).

The ΔP1 structure has a few important features in the α–β loop, namely the parallel β-sheet and the type III β-turn. The parallel β-sheet is not connected to the β-meander by backbone hydrogen bonding and is therefore not considered to be part of the head domain; however, they are kept in place via side chain–main chain hydrogen bonding between Asp90 of the first β-strand of the β-meander and the backbone amide of Ile57 (2.89 Å), as well as hydrogen bonding between Asp90, water 35 (2.61 Å, *B* factor of 17.76 Å^2^) and the backbone carbonyl of Lys55 (2.80 Å). Residues on the β-sheet proximal to the head domain Ile57 and Val58 are hydrophobic; Ile57 is buried inwards in the space between the α-helix and the head domain to perform hydrophobic interactions with many β-sheet residues, including Val88, Val96 and Leu98, as well as α-helix and α–β loop residues Ala47, Ile51 and Ile70 (Fig. 1 ▸*b*). In the context of an assembled pilus, this parallel β-sheet may be shifted to align its hydrophobic residues with those of the head domain and promote a larger hydrophobic surface that contributes to the interior of the pilus.

The α–β loop makes several important solvent interactions that aid in the stabilization of the observed architecture, such as in the first and second parallel β-strands which are bridged by water 37 (*B* factor of 7.88 Å^2^), which hydrogen-bonds to the side chain of Ser59 (2.85 Å) and the side chain of Asp69 (2.77 Å), resulting in the observed pitch between the strands. Ser59 and Asp69 also stabilize the type III β-turn, as the Asp69 side chain hydrogen-bonds to the side chain of Lys83 (2.83 Å), which is hydrogen-bonded via its backbone amide to the side chain of Ser59 (2.71 Å), creating an impressive web of powerful noncovalent bonds between these structural moieties (Fig. 1 ▸*c*). The cacodylic acid also makes hydrogen bonds which stabilize the α–β loop and the α1-C helix as its O atoms hydrogen-bond to the side chain of Thr73 (2.46 Å) and water 17 (2.94 Å, *B* factor of 20.59 Å^2^), which connects to the helix through a hydrogen bond to the side chain of Glu39 (2.75 Å). Thr73 has nonfavorable side-chain angles, which are likely to be due to hydrogen bonding to the cacodylic acid, which stabilizes the observed conformation suitable for crystallization; in the context of the typical environment of the pilin, the bacterial periplasm, this region may behave differently.

The β-meander is amphipathic; half (13/26) of the residues are hydrophobic and mostly occupy the space between the head domain and the α-helix (Fig. 1 ▸*b*) and the other half of the residues are polar, five of which are charged amino acids. These hydrophilic residues face away from the interior of the molecule and either make solvent contacts, contact side chains from the residues of neighboring β-strands or contact the symmetry mate. This is expected as they would face the solvated exterior of the assembled pilus. The hypervariable loops in between the β-strands have some important residues which support their conformation, such as Ser106 which hydrogen-bonds to the carbonyl of Gly103 using its backbone N atom (2.92 Å) to hold the β_2_–β_3_ loop in its conformation, and its side-chain hydroxyl makes a hydrogen bond to the main-chain carbonyl of Leu77 (2.87 Å) to bind it to the neighboring α–β loop. Lys104 is part of the β_2_–β_3_ hypervariable loop and displays unfavorable Ramachandran angles due to its hydrogen bonds to the carbonyl backbone of Gly80 (2.85 Å) and the symmetry mate Ser148 on the terminal carboxyl (2.86 Å), which stabilizes the C-terminal residues of the molecule. Arg119, at the beginning of the β_3_–β_4_ hypervariable loop, is the only residue with a bond-angle outlier, which is present in the N^ɛ^—C^ζ^—NH_2_ covalent bonds of the guanidinium group. This is likely to be caused by multiple hydrogen bonds that stabilize the atypical conformation; N^ɛ^ hydrogen-bonds to the side chain of Glu48 (2.88 Å), N^η1^ bonds to the other O atom of Glu48 (2.84 Å) and water 30 (2.90 Å, *B* factor of 21.03 Å^2^), while N^η2^ hydrogen-bonds to Gly94 (2.90 Å) and water 31 (2.89 Å, *B* factor of 24.31 Å^2^). This sequestration provides Arg119 with a side-chain *B* factor that is 10 Å^2^ lower than other arginine residues in this protein (8.4 Å^2^). Gly123 also makes an important hydrogen bond with its backbone carbonyl to water 33 (2.72 Å, *B* factor of 15.91 Å^2^), which along with the side chain of Trp125 hydrogen-bonds to the side chain of Glu48 (2.73 Å; 2.84 Å for Trp125) to bring the β_3_–β_4_ loop into contact with the α-helix (Fig. 1 ▸*d*). Glu48 is the first residue to resume the canonical α-helical backbone hydrogen-bonding geometry after the bend in the α1-C region (Section S1).

The D-loop is a conserved structural motif in *P. aeruginosa* pilins; however, the shape of the region differs due to changes in primary sequence. The conserved disulfide bond is formed by Cys127 in the fourth β-sheet and Cys145; however, Cys127 is observed to have electron density representative of an unbonded cysteine (∼20% occupancy), which is likely to be due to radiation-induced breakage of the disulfide bond (Stachowski *et al.*, 2020 ▸). There are some neighboring residues that stabilize the main-chain conformation near the disulfide; the backbone amide of Ile129 hydrogen-bonds to the carbonyl backbone of Pro146 (3.03 Å), and a proline from the loop immediately preceding the disulfide, Pro142, hydrogen-bonds to the main-chain amide of Cys145 using its backbone carbonyl (3.02 Å) (Fig. 1 ▸*e*). Prolines are expected to be rigid, hence the perceived conformational stabilization presented by these hydrogen bonds (Ge & Pan, 2009 ▸). However, despite a low side-chain *B* factor, Pro146 (10.1 Å^2^) is observed in an *endo*–*exo* conformational flip at a 50:50 ratio. Other important prolines in this region include Pro133, which induces the turn from the loop extending out of the final β-strand, and Pro138, which provides the kink in the type III β-turn observed in the D-loop region. The residues which make up this β-turn have the highest *B* factors of this moiety: Lys137, Pro138 and Asn139 have main-chain *B* factors of 14.1, 14.8 and 14.8 Å^2^, respectively. This region may be flexible to allow broad substrate recognition in the receptor-binding process as it is the tip of the receptor-binding domain, or it may simply be dynamic to allow conformational changes during the oligomerization process (Audette, Irvin *et al.*, 2004 ▸).

When looking at which regions have the highest *B* factors, ΔP1 is observed to have more thermal movement in the loop regions on the side of the protein opposite to the receptor-binding D-loop when compared with ΔK122 and Δ1244 (Fig. 1 ▸*f*). The loop which connects the two parallel β-strands in the α–β loop region consisting of Pro63–Thr66 has the highest *B* factors of the structural model (other than the N-terminal Ile25). Asp65 has a main-chain *B* factor of 22.8 Å^2^ and Thr66 has a poor RSR *Z*-score of 2.6%. Due to the poor electron density, the side chain of Asp65 is modeled in a nonfavorable conformation. Statistical outliers in both residues are likely to be due to the high thermal movement in this area as the region requires flexibility to be moved during pilus assembly. Another region that has high *B* factors is the β_1_–β_2_ loop from Asp90 to Thr95, which all have main-chain *B* factors above 10 Å^2^. Asn91 and Lys92 have particularly high *B* factors for this region (17.5 and 18.9 Å^2^, respectively), which is especially interesting as the residue which differs between P1 and 1244 is Lys92 (Gln in 1244), indicating that this flexible region may be of importance in the specificity and recognition of *P. aeruginosa* group Ia pili. Despite the homology in structure between the T4P of *P. aeruginosa*, different residues play discrete roles in dictating how the α-helix orients with the β-meander of the head domain, and of the important conformations of the connecting loop regions that provide the observed functional diversity in pili from different strains.

### 3.3. Insights from molecular-replacement solutions

**Figure 2:**
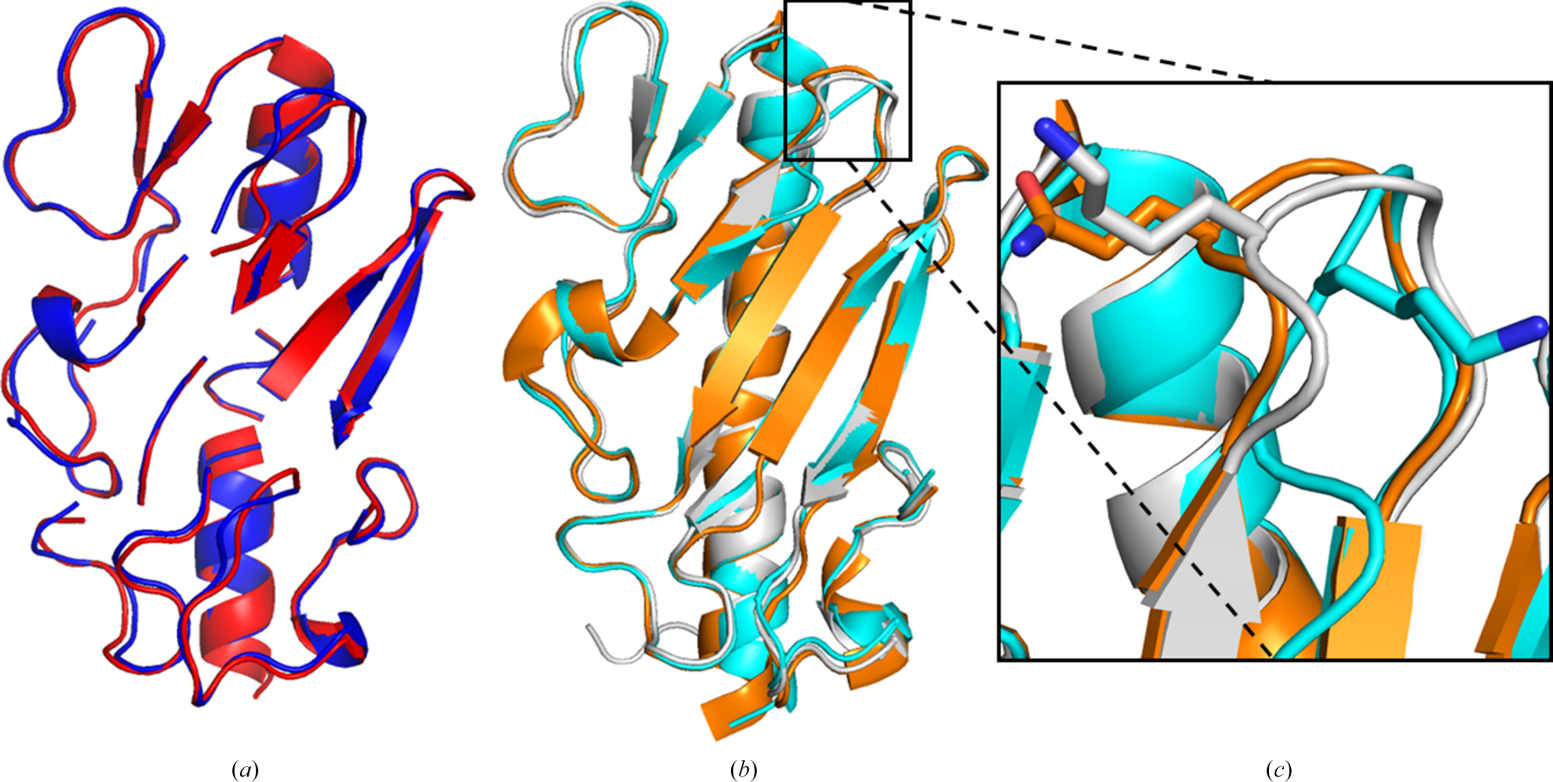
(*a*) *AlphaFold* and *RoseTTAFold* models of ΔP1 processed by the ‘process predicted model’ tool in *CCP*4*i*2 and used as search models for *Phaser* molecular replacement. The processed *AlphaFold* model was generated using *ColabFold* (version 1.5.2) and is colored red, while the processed *RoseTTAFold* model was generated using the *Robetta* server and is colored blue (Baek *et al.*, 2021 ▸; Mirdita *et al.*, 2022 ▸). (*b*) Superimposition of the unprocessed *AlphaFold* ΔP1 model (cyan) and the Δ1244 structure (orange; PDB entry 6bbk) with the ΔP1 structure (white). The region with the highest r.m.s.d. between the models is highlighted in (*c*); the differing side chain of residue 92 is shown and demonstrates the incorrect prediction of the positioning of Lys92 by *AlphaFold*. All images were generated using *PyMOL* version 2.5.0 (Schrödinger).

Initial attempts (in around 2003) at molecular replacement and experimental phasing of the ΔP1 structure were unsuccessful, and we were only *a posterori* aware of the structure of Δ1244 following the refinement and deposition of the current ΔP1 structure. Therefore, *AlphaFold* and *RoseTTAFold* models were generated using *ColabFold* (version 1.5.2) and the *Robetta* server, respectively, and employed as search models in *Phaser* (Baek *et al.*, 2021 ▸; Mirdita *et al.*, 2022 ▸). This allowed the predictive modeling of the residues remaining from the cleaved tag (ISEF) and the differing residue between ΔP1 and Δ1244 (K92Q), as well as a comparison between algorithms for producing models suitable for MR by *Phaser*. Initial *AlphaFold* and *RoseTTAFold* models were inadequate for MR; *Phaser* could not find a valid solution using the *RoseTTAFold* model and the values for the solution resulting from MR using the *AlphaFold* model were poor (Table 2 ▸). Using the ‘process predicted models’ tool in *CCP*4, preferable MR models were generated based on the regions determined to be in proximity based on the pLDDT score from the *ColabFold* output (Fig. 2 ▸*a*). Both *AlphaFold* and *RoseTTAFold* models were separated into five domains to be used as separate search models, consisting of domain 1 (Ser14–Ala18), domain 2 (Ser2–Val13, Gly79–Val90 and Ile105–Ser124), domain 3 (Leu19–Phe48), domain 4 (Thr49–Val64 and Val75–Leu78) and domain 5 (Thr65–Leu74 and Thr92–Lys104), where Ile1 and Ile91 were removed from both search models and Ser2 and Glu3 were removed only in the *RoseTTAFold* model. These processed search models provided solutions via *Phaser* with improved scores compared with their unprocessed counterparts; however, the solution obtained from the processed *RoseTTAFold* model was less reliable than the unprocessed *AlphaFold* model. Further, when the Δ1244 structure was used as a MR search model, *Phaser* provided a successful result with an *R*_free_ of 0.482; however, the processed *AlphaFold* model was the optimal search model, providing an output solution with an *R*_free_ of 0.453.

Comparing the refined ΔP1 structure with the MR search models, the trend in the *Phaser* output parameters is reflected in the main-chain r.m.s.d. of search models aligned with the ΔP1 structure; the *RoseTTAFold* model has a high r.m.s.d. of 7.041 Å. The alignment of ΔP1 with the *AlphaFold* and Δ1244 models are very similar: they have r.m.s.d. values of 0.513 and 0.616 Å, respectively (Fig. 2 ▸*b*). Despite the higher r.m.s.d. of the Δ1244 model, there are some areas where the structure of ΔP1 more closely matches the structure of Δ1244, most notably in the hypervariable loop near the single non­homologous residue Lys92 (Fig. 2 ▸*c*). This region has high *B* factors in the ΔP1 structure, which may be why the *AlphaFold* predictive model was not successful in modeling the placement of the side chain. The similar positioning of the residues in ΔP1 and Δ1244 is interesting as there are no observable intermolecular hydrogen bonds holding this region of the loop in position, suggesting a role in pilus differentiation/recognition between different strains of *P. aeruginosa*. Notably, when the *AlphaFold* model was processed via the ‘process predicted model’ functionality in *CCP*4*i*2 the solution was improved compared with using the full-length model for MR. The hypervariable loop region containing Lys92 may have needed to be processed to separate the search model into distance-related domains, allowing *Phaser* to more effectively use this domain as a search model, and demonstrates the effectiveness and prudence of processing AI-predicted models to produce superior MR search models.

### 3.4. Sequence alignment is not indicative of structural alignment in type IV pilins

*PISA* analysis did not detect a dimerization interface for ΔP1 (Section S2); however, it was useful for finding T4P with structural homology based on similarities in their crystallo­graphic interfaces (Krissinel & Henrick, 2007 ▸). The proteins with the highest *Q*-scores were the crystallographic interfaces of triclinic and monoclinic crystal structures of ΔK122 (*Q*-scores of 0.436 and 0.428, respectively; Audette, Irvin *et al.*, 2004 ▸), the truncated minor pilin PilV pilin from *Neisseria meningitidis* strain C8013 (*Q*-score 0.425); PDB entry 5v0m; S. Kolappan & L. Craig, unpublished work), the full-length PAK pilus solved using cryo-EM (*Q*-score 0.379; PDB entry 5vxy; Wang *et al.*, 2017 ▸) and the truncated pilus from *Shewanella oneidensis* strain MR-1 (*Q*-score 0.372; PDB entry 4d40; Gorgel *et al.*, 2015 ▸). These identified pilin proteins were used for comparative analyses.

*Clustal Omega* alignment of sequences of T4aP from *P. aeruginosa* (P1, K122, 1244, PAK and 110594), *N. gonorrhoeae* strain C30 (Ng_C30), *N. meningitidis* strain C8013 (Nm_C8013), *S. oneidensis* strain MR-1 (So_MR-1) and the T4bP from *Vibrio cholerae* strain RT4236 (Vc_RT4236) was performed to compare the amino-acid sequences of pilins with similar structures and interfaces. The alignments start from their α1-N domain as the signal sequence is cleaved prior to pilus assembly and is not present in any of the high-resolution structures of these T4P; an additional alignment was performed using the sequences of the head domain of each T4P. A sequence-alignment matrix for comparing percentage of alignment between structurally related T4P demonstrates that the T4P from *P. aeruginosa* are most closely related to each other, with the exception of K122, which is most closely related to Ng_C30 (Table 3 ▸, Supplementary Fig. S3). In comparing the sequence alignments via the alignment matrix, it is evident that the α1-N helix is the region of highest sequence homology between the pilins, an established trend for the T4P from *P. aeruginosa*, which interestingly appears to cross phylogenies. The trends in percentage of sequence alignment are approximate when comparing sequence alignments of full pilins with those without the α1-N helix; for brevity, only the alignments from sequences of the full pilins will be discussed. When comparing group Ia pilins with the other pilins it becomes evident that they share the highest sequence homology with group II pilins (PAK and K122), with the group V pilin PilA from *P. aeruginosa* strain 110594 (110594) having a similar sequence alignment (34.1%) to T4aP from other species (Ng_C30 at 36.1%) (Nguyen *et al.*, 2010 ▸). However, the difference in homology between the group II pilins in this study is lower than the homology between group Ia and II pilins, with an alignment of 34.6% between K122 and PAK versus an alignment of 43.1% between PAK and P1. K122 also shows the highest homology to Ng_C30, at 44.7% alignment, versus any of the other *P. aeruginosa* pilins, the highest alignment score of the matrix. In comparing the group V pilin 110594 with the other *P. aeruginosa* pilins, K122 is the most homologous, at 40.9% alignment, while group Ia pilins share 34.1% alignment, which is slightly higher than the alignment of So_MR-1 at 30.3%. This indicates that although group I and group II pilins are of higher homology to each other based on sequence alignment, the interspecies homology of T4aP is comparable to the inter-strain homology of T4aP from *P. aeruginosa* (Natalini *et al.*, 2023 ▸; Beck *et al.*, 2012 ▸).

The sequence alignment and conservation of the T4P determined with *Clustal Omega* shows the expected results; the α1-N region has high sequence conservation and the areas in which the α1-C sequence differs (such as Arg35 in P1 and Glu35 in K122) are simply residue-swapped for the side-chain interactions that occur between the α-helix and the α–β loop (*i.e.* Ser75 in P1 and Lys80 in K122) such that an intermolecular hydrogen-bond holding these regions is maintained (Supplementary Fig. S4). Lys44 is a highly conserved residue in the α1-C region as it appears to be an important residue for maintaining head-domain pilin–pilin interactions in the assembled pilus (Neuhaus *et al.*, 2020 ▸; Wang *et al.*, 2017 ▸; Ochner *et al.*, 2024 ▸). The only areas with no sequence alignment for a stretch greater than eight residues seen in P1 and 1244, compared with the other pilins, are the first part and last part of the α–β loop from residues Ile51 to Ala61 and residues Asp79 to Gln87. These regions include some important residues for maintaining the shape of the ΔP1 pilin (Section 3.2), including the hydrophobic interactions of the α–β loop (Fig. 1 ▸*b*) and the observed hydrogen-bonding network (Fig. 1 ▸*c*). Homologous residues in the β-sheets include Thr101 and Gly112, which in the case of ΔP1 create a bend in the β-sheet between the second and third β-strands. Trp125 is one of few highly conserved residues in the C-terminal half of *P. aeruginosa* pilins and is shown to hydrogen-bond to Glu48 (Fig. 1 ▸*d*). There is a conserved polar residue capable of hydrogen-bonding in other T4P, indicating that this interaction is likely to be important in maintaining the proximity between the α-helix and the β-meander in these pilins, and it may also influence the bend of the α-helix. The residues after the last β-strand that start the D-loop display low sequence conservation, as the only homologous residues in the D-loop are Cys127, Pro142 and Cys145; they maintain the main-chain conformation that is essential for T4P binding (Audette, Irvin *et al.*, 2004 ▸).

Analysis of structural alignment was performed via *Pro­SMART* to ensure that conformational flexibility was considered when comparing closely related T4P structures (Nicholls *et al.*, 2014 ▸). In aligning the *AlphaFold*-predicted model with the ΔP1 structure, the average Procrustes r.m.s.d., the average Flexible score, the global r.m.s.d. and the Best Fragment score were all higher than when ΔP1 was aligned with the Δ1244 structure (Table 4 ▸). Knowing that there are a number of well refined pilin structures that are likely to be employed in the training sets of AI model-prediction suites, including *AlphaFold* and *RoseTTAFold*, and that the best MR search model for *Phaser* was one processed prior to use, these data suggest that further improvements to prediction algorithms that incorporate such conformational flexibility could lead to better search-model predictions.

As expected, alignments with a low average Procrustes score often had proportionally low average Flexible, global r.m.s.d. and Best Fragment scores. There was an unusual relationship between *Clustal Omega* sequence alignment and structural alignment to ΔP1; there were many comparisons made in which the sequence alignment was lower than for another pilin but the average scores from *ProSMART* were relatively improved. This is most apparent when comparing the alignment of 43.1% for ΔK122 with the alignment of 25.5% for Nm_C8013, which has the lowest alignment with ΔP1 of the T4aP sequences compared; however, the average Flexible score for Nm_C8013 is 1.14, superior to the scores of 1.24 and 1.62 for *P. aeruginosa* pili ΔK122 and Δ110594, respectively. Although most scores were slightly higher when aligning the PAK pilin with the ΔP1 structure versus the alignment of ΔPAK, as caused by the lack of the α1-N helix in the ΔP1 structure, the Best Fragment score improved for the full-length PAK pilin; the full-length PAK pilin was recognized as having a similar interface to ΔP1 via *PISA*, whereas ΔPAK did not have the same *Q*-score, indicating that some regions alter when the hydrophobic α1-N is not present, demonstrating the value of solved T4P structures with an intact α1-N helix. Other than Δ1244, the T4aP that most closely resembles ΔP1 of the compared structures is ΔPAK, whereas the least homologous is Δ110594. Pilins from different species such as Ng_C30 and Nm_C8013 have lower scores from *ProSMART* than Δ110594, thus indicating the possibility of higher structural variability of T4aP between strains of *P. aeruginosa* than the variability between T4aP from different species. Also, the comparison of ΔP1 with the T4bP ΔVc_RT4236 provides further evidence that group V pilins are more similar in structure to T4bP than to group I and II pilins, as sequence-alignment scores and previous studies indicate (Nguyen *et al.*, 2010 ▸); however, structural alignment scores for ΔVc_RT4236 and Δ110594 to ΔP1 are also similar despite their large differences in sequence alignment with P1.

### 3.5. Type IVa pilins are differentiated by their α–β loop

**Figure 3:**
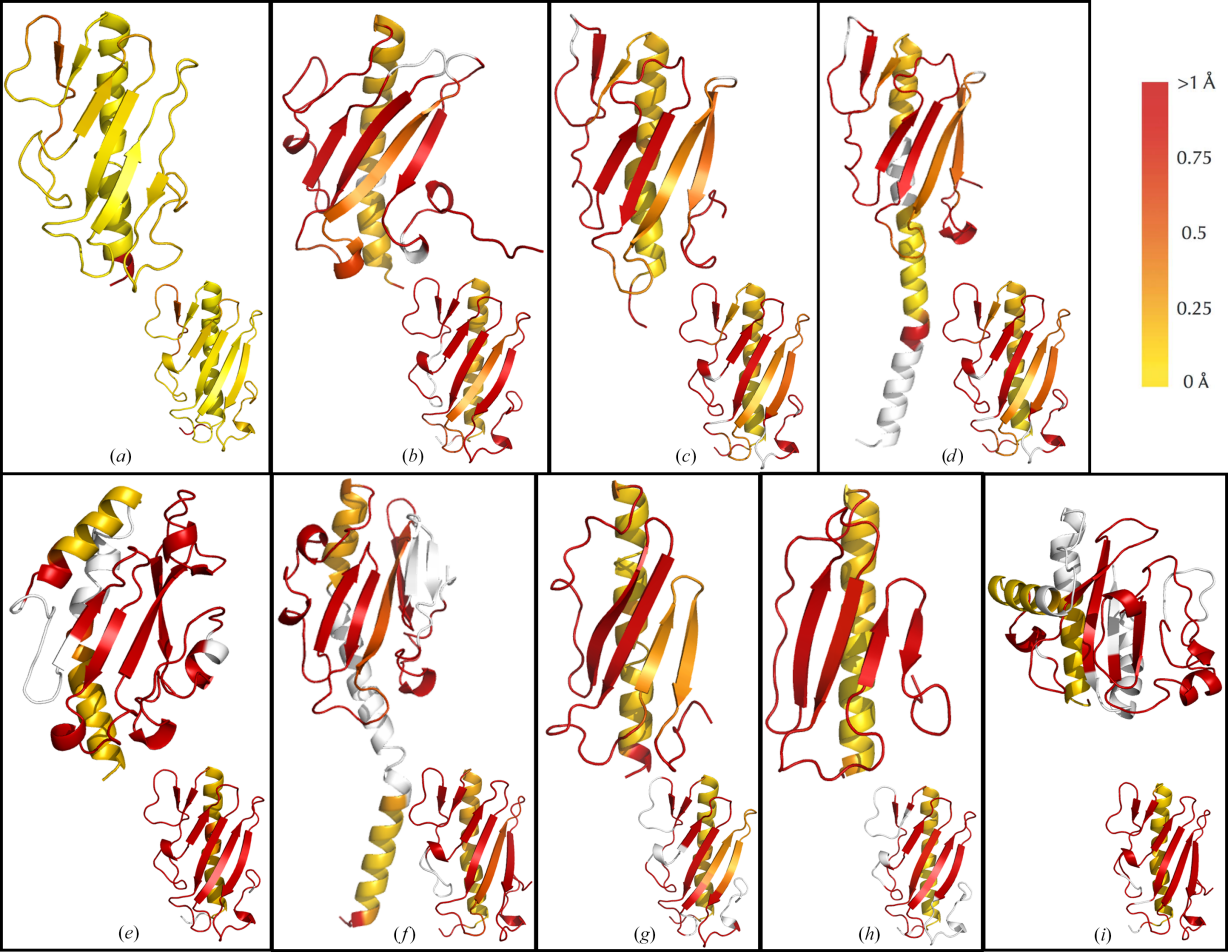
*ProSMART* conformation-independent structural alignment of ΔP1 with similar T4P, colored by the average Flexible score as per the legend in the top right of the figure based on the r.m.s.d. between backbone atoms of the aligned structures (Nicholls *et al.*, 2014 ▸). Regions colored white are not present in the aligned protein and therefore are not comparable; the ΔP1 structure is to the bottom right of each compared pilin and is colored by Flexible scoring for the respective alignment. The structures aligned with ΔP1 are (*a*) Δ1244 (PDB entry 6bbk), (*b*) ΔK122 (PDB entry 1qve), (*c*) Δ110594 (PDB entry 3jzz), (*d*) ΔPAK (PDB entry 1dzo), (*e*) PAK (PDB entry 1oqw), (*f*) Nm_C8013 (PDB entry 5v23), (*g*) So_MR-1 (PDB entry 4d40), (*h*) Ng_C30 (PDB entry 2hi2) and (*i*) Vc_RT4236 (PDB entry 1oqv).

In viewing the *ProSMART* alignment between the ΔP1 and Δ1244 structures, the structural homology is extremely high; however, the single residue difference between these pili affects the conformation of the α–β loop rather than influencing the main-chain conformation of the residues in the first hypervariable loop where residue 92 resides (Fig. 3 ▸*a*). Asp65–Thr73 feature residues with the highest side-chain *B* factors of ΔP1. They may be different merely due to differences in crystal contacts holding these regions in place; however, the Flexible parameter in *ProSMART* indicates that this region has a discretely different conformation in ΔP1 compared with Δ1244.

There are many structural distinctions observed in comparing ΔP1 and *P. aeruginosa* pilins from other groups (Section S3). The α1-C helix is the only region that shares good alignment based on the Flexible score when aligning ΔP1 and the group II pilins ΔK122 and ΔP1 (Fig. 3 ▸*b*). In comparing ΔP1 and ΔK122, the third β-strands of the two pilins are moderately aligned from Ile110 to Arg118 in ΔP1; otherwise, all regions have poor alignment. ΔP1 and ΔPAK appear to be highly similar upon structural alignment; however, Flexible scoring shows that there are major differences in the conformation of the pilins starting in the α–β loop region due to residues in ΔPAK that are not present in ΔP1 (Fig. 3 ▸*c*). The structures are moderately similar in the third and fourth β-strands; however, the D-loop regions of the proteins are dissimilar, owing to a region in ΔP1 that is not present in ΔPAK. Comparing full-length PAK with ΔP1, the head domain aligns in an identical fashion as ΔPAK but the α-helix no longer aligns with the same residues; there is a gap in the α1-C helix of PAK that is recognized as not being present in ΔP1 despite being recognized as highly aligned in ΔPAK (Fig. 3 ▸*d*). Instead, a region in the α1-N helix of PAK is predicted to conformationally align with the α1-C helix of ΔP1. This provides evidence that structures of pilins with the α1-N region may provide more accurate comparisons of the α-helical regions of pilins. *PISA* analysis may recognize different aspects of the interfaces present in the pilin structures with α1-N helices, but they do not often make a difference in the structure of the rigid head domain and are not likely to be essential for relating *in vivo* functions from structures of T4aPs.

Group Ia and group V pilins are structurally distinct; however, when aligning ΔP1 and Δ110594 there are no regions of ΔP1 that are not recognized as fragments in Δ110594 based on Flexible scoring: the group V pilin merely has extra regions which differentiate it (Fig. 3 ▸*e*). Unlike the alignments with the other *P. aeruginosa* pilins, ΔP1 does not align well with Δ110594 throughout the whole α1-C helix, and all other regions in ΔP1 align poorly with Δ110594. Despite the complex secondary-structural regions in the hypervariable loops and the differences in the β-sheet topology of Δ110594, *ProSMART* recognizes that these regions have some structural similarity despite displaying poor sequence and structural alignment overall.

*ProSMART* alignment of ΔP1 with T4aP from other species shows similarities in alignment with the group II pilins analyzed. Of the three observed, Ng_C30 has the lowest structural alignment scores despite having optimal sequence alignment with ΔP1, which is likely to be due to the additional regions in the head domain of Ng_C30 and the smaller size of the head domain of the other pilins ΔNm_C8013 and ΔSo_MR-1. The structure of Ng_C30 aligns with ΔP1 in a similar fashion as PAK, where the α1-N region of Ng_C30 that is not present in the ΔP1 structure is instead aligned with the initial α1-C residues (Fig. 3 ▸*f*), once again demonstrating the utility of structures with the α1-N region. The only other region with moderate structural alignment is the third β-strand; all regions have poor alignment with ΔP1. There is one motif in the D-loop of Ng_C30 from Val125 to Asp138 which appears to be unique to this T4aP and is not present in ΔP1. ΔNm_C8013 and ΔSo_MR-1 both have similar crystallographic interfaces based on *PISA* analysis, and in structurally aligning with ΔP1, the α1-C of ΔNm_C8013 and ΔSo_MR-1 are highly homologous up until the residues that cause the kink in the α-helix. ΔP1 is somewhat conformationally distinct from the other two pilins in this region, which appear to have canonically straight α1-C helices (Figs. 3 ▸*g* and 3 ▸*h*). Some regions in ΔP1 are not present in ΔNm_C8013 and ΔSo_MR-1, and the difference in the size of these absent regions when aligning with ΔP1 is likely to contribute to the lower structural alignment with ΔSo_MR-1 than with ΔNm_C8013 despite the opposite being true for their respective sequence alignments. The third and fourth β-strands of ΔNm_C8013 align moderately well with ΔP1; however, ΔSo_MR-1 has poor structural alignment with ΔP1 for the remainder of the protein. Structural similarity of the third and fourth β-strands appears to be a trend of pilins that have improved scores, as the global r.m.s.d. of the alignment between ΔP1 and ΔNm_C8013 is the third lowest of those compared; similar alignments occur with ΔK122, PAK and Ng_CR30 (Figs. 3 ▸*b*, 3 ▸*d* and 3 ▸*f*). Interestingly, ΔNm_C8013 and ΔSo_MR-1 do not have a receptor-binding D-loop. The ΔSo_MR-1 pilin is thought to be important to the species for extracellular electron-transfer pathways and is not primarily used for cellular adhesion. However, the minor pilin ΔNm_C8013 has not been well characterized; based on these structural comparisons and information on homologous minor pili it is also likely to have alternative functions.

The structural comparison of ΔP1 with the T4bP ΔVc_RT4236 shows that they align similarly to the manner in which ΔP1 aligns with Δ110594, again further supporting the structural similarity of group V pilins, and by extension group III pilins, to T4bP (Fig. 3 ▸*i*; Nguyen *et al.*, 2010 ▸). ΔP1 varies significantly from ΔVc_RT4236, with only the α1-C helix of ΔP1 scored as aligning well. In ΔP1 there are only a few residues that are recognized as not being aligned with fragments from ΔVc_RT4236, and they include the α–β loop residues Leu78–Gly80 and Gly103 on the β_2_–β_3_ loop, which coincidentally perform intermolecular interactions. Although these are just a few residues of many that are not sequence-aligned between ΔP1 and ΔVc_RT4236, the divergent evolution which causes the deletion of interacting residues such as these are an example of how the observed structural diversity in T4Ps arose and continues to evolve.

A kink in the α1-C helix was observed in the structure of ΔP1 (Fig. 1 ▸*a*, Supplementary Fig. S1); in comparing the structures of pilins via *ProSMART* this bend was observed to vary in pitch in different pilins (Fig. 3 ▸). This bend in the α1-C helix is common in T4P, yet only the severe kink in the α1-N region is often discussed in the literature; it is seen in cryo-EM reconstructions of the PAK and *N. gonorrhoeae* strain C30 pili and therefore is not caused by crystallographic packing interactions (Wang *et al.*, 2017 ▸). We propose that it allows cushioning between the two moieties of the head domain as there are bulky hydrophobic side chains on the α1-C helix and the β-meander that populate the interior space between the two domains. This region excludes water, which contributes to the rigid rod-like behavior of the assembled T4P (McCallum *et al.*, 2019 ▸). The kink is shallower in group Ia versus group II pilins, potentially due to the presence of a hydrophilic residue, Glu48, in the group Ia pilins, which is one of few nonconserved residues in the α1-C helix. No fragments aligned with the bent region from Ser41 to Lys44 in ΔP1 when comparing with the group V pilin, while the minor pilin ΔNm_C8013 and the extracellular electron-transfer pathway pilin ΔSo_MR-1 have a straight helix α1-C region and have different roles to the other T4P shown. This kink may serve a role akin to the α1-N helix break that is conserved in many T4P from residues 15 to 23, which ‘melts’ to accommodate the assembly of the pilus by aiding movement of the helix in the hydrophobic interior (Ochner *et al.*, 2024 ▸; Wang *et al.*, 2017 ▸). However, as it has a different kink depending on the pilin and it is not present in the pilins with alternative roles, the bend in the α1-C may provide a mechanical signal for pilus attachment and/or depolymerization, straightening upon either event as the mechanism for function.

The architecture of α–β loops appears to best distinguish pilins from each other; pilins of lower homology appeared to have additional or missing α–β loop regions, as was confirmed by the *Clustal Omega* alignment results (Supplementary Fig. S4), and those close in sequence homology in many instances were seen to have higher Flexible scoring and therefore are conformationally distinct, despite appearing to be similar at first glance. As mentioned, this region in ΔP1 contains the residues with the highest *B* factors of the protein; however, *ProSMART* recognizes it as being sufficiently unique to not align well with the other α–β loops of pilins. The D-loops of the pilins were also conformationally distinct despite previous studies demonstrating main-chain homology, indicating that there is enough rigidity in the regions for each to be distinct and therefore differentiated from each other. Interestingly, the group Ia pilins studied are the only pilins compared which have prolines bordering both cysteines in the D-loop, and Pro146 performs *endo*–*exo* conformational flipping; this may provide the rigidity required for the observed diversity in the main-chain conformation but allows some rotamer movement to accommodate the dynamics required for the function of this region (Fig. 1 ▸*e*). This provides further evidence for the D-loop region in aiding the mechanism of receptor-binding specificity and recognition, but also adds the α–β loop as an additional distinguishing factor for T4aPs.

### 3.6. Differences in self-assembly of type IV pilins

Self-assembly of the ΔP1 pilus was assessed in a manner previously performed for the ΔK122 pilin (Petrov *et al.*, 2013 ▸; Lento *et al.*, 2016 ▸). Understanding pilin oligomerization *in vitro* is important in the development of anti-infective therapeutics targeting the *P. aeruginosa* T4P and applications of pilin-based PNTs as drug-delivery vehicles (Audette *et al.*, 2019 ▸). *PISA* analysis of the ΔP1 structure predicted that all potential interfaces have no role in complex formation and are a result of crystal packing only (Section S2 and Supplementary Fig. S2). The crystallographic interface of ΔP1 is most similar to that of ΔK122 despite the additional dimeric interface of ΔK122, both of which are not in the same configuration as the ΔP1 crystallographic interface (Lento *et al.*, 2016 ▸; Audette, Irvin *et al.*, 2004 ▸). The crystallographic interface of the ΔK122 pilin stacks the β-meander head groups of the two symmetry-related molecules, while the dimeric interface is from the α–β loop to the bottom of the α1-C helix. Nevertheless, ΔK122 self-oligomerizes into PNTs (Audette, van Schaik *et al.*, 2004 ▸; Petrov *et al.*, 2013 ▸; Lento *et al.*, 2016 ▸). Therefore, an analysis of the self-assembly of ΔP1 was performed via SEC-MALS to separate any oligomers which may form by size, accurately determine their molecular weight (MW) and gain and understanding of the general shape of the eluted species.

**Figure 4:**
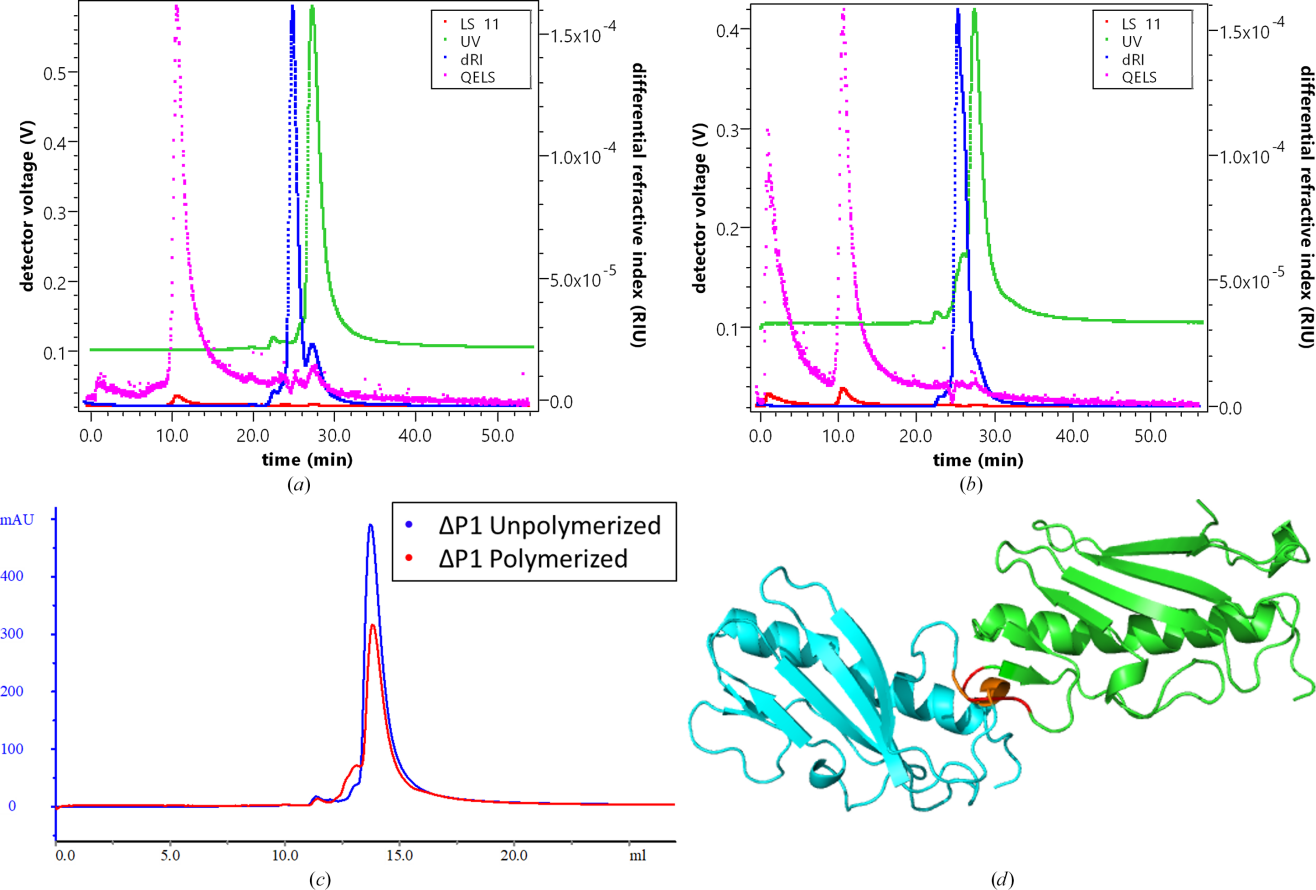
Oligomerization analysis of ΔP1 using SEC-MALS and a three-start helical model. MALS analysis of (*a*) unoligomerized and (*b*) oligomerized ΔP1 samples after performing inline SEC at a flow rate of 0.5 ml min^−1^. Images were created using the *ASTRA* software suite version 6.0 (Wyatt). (*c*) Overlaid UV_280 nm_ chromatograms from both experimental runs. (*d*) A model for the assembly direction of the pilus using the ΔP1 pilin structure based on a three-start helical assembly (Petrov *et al.*, 2013 ▸; Craig *et al.*, 2006 ▸). Monomers are shown in green and cyan, with clashing residues in red and orange; the direction of pilin oligomerization would be from the cyan molecule to the green molecule.

When analyzed by SEC-MALS, ΔP1 was observed to be monomeric (Fig. 4 ▸*a*). This is unlike ΔK122, which displays a monomer–dimer equilibrium when in the same buffer (Petrov *et al.*, 2013 ▸; Lento *et al.*, 2016 ▸). Furthermore, when oligomerization was attempted under conditions demonstrated to be optimal for ΔK122, including the hydrophobic catalyst MPD, ΔP1 was observed to only partially dimerize and does not indicate the presence of any polymers (Fig. 4 ▸*b*). On overlapping the UV_280 nm_ chromatograms a slight increase in the dimer peak becomes visible (Fig. 4 ▸*c*). Some useful biophysical parameters were obtained from MALS analysis; the MW of the monomer peak from the unpolymerized ΔP1 sample is calculated to be 14.81 kDa (±0.909%; expected MW 13.03 kDa) and the small shoulder corresponding to the dimer in the polymerized sample has a MW of 21.93 kDa (±3.738%; expected MW 26.06 kDa). The difference in MW for the monomer can be attributed to solvent hydration; however, the MW difference for the dimer is likely to be due to its small proportion of the LS signal causing inaccuracy in the MW estimation (9.1% of the mass fraction). The small light-scattering (LS) peaks corresponding to the UV_280 nm_ signals of the monomer and dimer in both experiments hindered accurate calculation of the radius of hydration and radius of gyration. The LS peak with a high quasi-elastic light-scattering (QELS) signal seen in both experiments has no UV_280 nm_ signal and is considered to be an anomaly as it elutes in the void volume of the SEC column.

To conceptualize why ΔP1 does not oligomerize *in vitro* in the same manner as ΔK122, the structure of the ΔP1 pilin was modeled in a three-start helical assembly, the configuration that best describes the way in which pilin monomers assemble into a polymer, as originally characterized by cryo-EM experiments on the assembled Ng_C30 pilus (Fig. 4 ▸*d*; Petrov *et al.*, 2013 ▸; Craig *et al.*, 2006 ▸). Regarding the positioning of the monomers, there are clashes that occur between the α–β loop residues Pro63–Thr66 of one monomer and the D-loop residues Pro138–Ala141 of the other monomer; these regions are not pronounced in ΔK122. *PISA* provided further insight into why ΔP1 does not polymerize in the same fashion as ΔK122; an interface for oligomerization could not be found. However, the region in the α–β loop that is clashing has the highest *B* factors for ΔP1, and one could imagine that it might change conformation in forming polymers; perhaps minor pilins and/or other chaperones are required to induce the proper fold in both regions for P1 oligomerization. There are also several other factors that could influence the *in vitro* oligomerization of ΔP1, such as the presence of particular buffer conditions, a hydrophobic trigger molecule or TfpO glycosylation of the C-terminal serine to properly oligomerize *in vitro*. We are currently exploring these potential experimental conditions for the *in vitro* oligomerization of the ΔP1 pilin.

## 4. Conclusion

The high-resolution structure of ΔP1 allowed a thorough structural analysis of the protein, the first such characterization of a group I pilin from *P. aeruginosa*. The structure was solved 20 years after diffraction data collection, largely due to advancements in structural prediction software and other complementary software which allowed the creation of an optimal search model for MR. However, *AlphaFold* might not have created such a reliable model if the truncated structure of PilA from strain 1244 had not been uploaded to the PDB (Y. Shen, Y. Nguyen, A. Guarne & L. L. Burrows, unpublished work), reinforcing the importance of uploading data to repositories to allow more robust training sets for predictive modeling. The predicted ΔP1 model from *AlphaFold* was superior to that from *RoseTTAFold*; we suggest that the *Robetta* server could include an option to provide an output suitable for MR, as even when H atoms were deleted from the model it would not align well with the solved ΔP1 structure (Table 2 ▸), and although the *PyMOL* command to remove hydrogens is simple there are minor differences which prevents the output from being a suitable MR search model. Alternatively, the ‘process predicted model’ tool in the *CCP*4*i*2 GUI could provide the option to remove hydrogens from a search model.

The ΔP1 pilin displays higher homology to some distantly related pili than to ΔK122, which is further supported by the differences in their propensity for dimerization in solution and their ability to detectably oligomerize in the presence of a hydrophobic trigger molecule. *PISA* analysis indicates that there is potential for ΔP1 to dimerize, which supports our SEC-MALS observations; however, the dimerization and crystallographic interfaces are likely to be different, and the ΔP1 dimerization interface may be different from the ΔK122 dimerization interface seen in mass-spectrometry experiments (Lento *et al.*, 2016 ▸; Fig. 4 ▸*d*, Supplementary Fig. S2). The hydrophobic trigger molecule MPD allowed some dimerization of ΔP1, but not oligomerization, and we propose that the structural differences observed in the α–β loop and the D-region play a significant role, especially considering that the three-start helical model of pili generation shows that these two regions clash when applied to the ΔP1 structure. Mass-spectrometry experiments exploring the dynamics of these regions in ΔP1 are ongoing.

Comparing the structure of ΔP1 with a variety of T4P provided a context for the distinctions between group Ia pilins and other groups of *P. aeruginosa* pilins, as well as pilins from different species. Sequence and structural comparisons identified that ΔP1 and ΔPAK are most similar in structure, while ΔK122 has greater homology to ΔNg_C30 than to either of these *P. aeruginosa* pilins. These findings suggest that a more nuanced, structural grouping of *P. aeruginosa* pilins to align with the current genetic grouping of pilins could be beneficial. The α–β loop may be of functional importance as it differs dramatically between closely related pilins; the structures of ΔP1 and Δ1244 only differ by one residue and were most structurally distinct in this region (Fig. 3 ▸*a*). All other comparisons identified the α–β loop and the D-loop as having residues that were not present or greatly differed in conformation, while the α-helix was highly conserved in every structure. Targeting the D-loop is likely to remain the best approach for both vaccine and antivirulence drug development against T4P from *P. aeruginosa*; however, care should be taken to prevent T4P from other bacteria not being targeted as well to prevent destruction of the lung microbiome (Beck *et al.*, 2012 ▸; Natalini *et al.*, 2023 ▸). High-resolution structural information on *P. aeruginosa* T4aP from all groups will help to ascertain the structure–function relationship of the diverse pilins in order to better understand how they bind receptors, how they relay binding signals and how they self-assemble for the formation of PNTs in the development of drug-delivery vehicles. The ΔP1 structure is one piece in a puzzle to dismantle the diverse weapons of the human pathogen *P. aeruginosa*.

## Supplementary Material

PDB reference: truncated P1 pilin from *Pseudomonas aeruginosa*, 8v7p

Further details and Supplementary Figures. DOI: 10.1107/S205979832401132X/gri5002sup1.pdf

## Figures and Tables

**Table 1:** Data-collection, processing and refinement statistics Values in parentheses are for the outer shell.

Diffraction statistics	
Diffraction source	ALS beamline 8.3.1
Wavelength (Å)	1.0236
Temperature (K)	100
Space group	*P*2_1_
*a*, *b*, *c* (Å)	22.45, 54.85, 37.77
α, β, γ (°)	90, 96.15, 90
Mosaicity (°)	0.79
Resolution range (Å)	54.85–1.30 (1.33–1.30)
No. of observations	89356 (3512)
No. of unique reflections	22344 (1309)
Completeness (%)	99.5 (99.2)
Multiplicity	4.0 (2.7)
〈*I*/σ(*I*)〉	14.2 (3.9)
*R*_p.i.m._	0.069 (0.355)
*R*_merge_	0.141 (0.498)
CC_1/2_	0.988 (0.639)
Overall *B* factor from Wilson plot (Å^2^)	7.6
Refinement statistics	
Resolution range (Å)	54.85–1.300 (1.334–1.300)
Completeness (%)	99.5
No. of reflections, working set	21642 (1603)
No. of reflections, test set	682 (39)
Final *R*_cryst_	0.1306 (0.131)
Final *R*_free_	0.1738 (0.176)
No. of non-H atoms	
Protein	957
Ligand	5
Water	216
R.m.s. deviations	
Bond lengths (Å)	0.0100
Angles (°)	1.9165
Average *B* factors (Å^2^)	
Overall	14.43
Protein	11.525
Ramachandran plot	
Most favored (%)	97.5
Allowed (%)	2.5

**Table 2:** *Phaser* molecular-replacement statistics of solutions for the ΔP1 pilin structure when using various search models

	Refined LLG	*R*_free_	TFZ	Main-chain r.m.s.d.† (Å)
*AlphaFold*, full	347.6	0.507	10.9	0.513
*AlphaFold*, processed‡	1145.6	0.453	19.0	
*RoseTTAFold*, full	Failed			7.041
*RoseTTAFold*, processed	112.1	0.565	6.2	
Δ1224	593.7	0.482	16.2	0.616

† Root-mean-square deviation distance between main-chain atoms of the MR search model when aligned with the refined ΔP1 structure using the *PyMOL* version 3.0.0 align command (Schrödinger).

‡ Processed models refer to the output from the ‘process predicted model’ tool from *CCP*4*i*2 (Agirre *et al.*, 2023 ▸).

**Table 3:** Sequence similarity (%) between similar type IV pilins†‡

	Group Ia	PAK	110594	K122	Ng_C30	Nm_C8013	So_MR-1	Vc_RT4236
Group Ia	—	43.1	34.1	38.7	36.1	25.5	32.0	14.4
PAK	28.7	—	37.3	34.6	31.8	23.8	29.0	15.8
110594	19.6	21.6	—	40.9	36.6	29.4	30.3	19.2
K122	24.3	26.4	29.3	—	44.7	27.3	28.6	14.7
Ng_C30	21.0	20.2	23.9	37.8	—	27.2	26.5	17.1
Nm_C8013	17.0	11.1	12.0	6.3	9.8	—	31.0	16.2
So_MR-1	14.6	2.3	13.0	15.4	14.3	18.8	—	21.6
Vc_RT4236	9.89	14.3	19.6	14.7	17.1	13.3	27.4	—

† Group Ia represents both ΔP1 and Δ1244, which are 99.3% identical; Ng_C30 is the major pilin PilE from *N. gonorrhoeae* strain C30; Nm_C8013 is the minor pilin PilV from *N. meningitidis* strain C8013; So_MR_1 is the major pilin PilD from *S. oneidensis* strain MR-1; Vc_RT4236 is the T4bP from *V. cholerae* strain RT4236.

‡ Sequence similarities for alignments of full-length pilins (minus signal peptides) and solely the head domain are displayed in the upper right half and lower left half of the table, respectively.

**Table 4:** Structural (*ProSMART*) and sequence (*Clustal Omega*) alignment values comparing ΔP1 with other type IV pilin structures

	AF ΔP1†	Δ1244	ΔK122	PAK	ΔPAK	Δ110594	Ng_C30	Nm_C8103	So_MR-1	Vc_RT4236
Residues aligned (total residues)	124 (124)	124 (124)	116 (126)	116 (144)	116 (120)	119 (148)	115 (150)	98 (98)	89 (89)	118 (171)
PDB sequence alignment/full length‡ (%)	100.0/100.0	96.0/99.3	10.3/38.7	22.4/43.1	25.47/43.1	10.1/34.1	13.9/36.1	10.2/25.5	7.87/32.0	8.47/14.4
Local alignment r.m.s.d. (Å)	0.508	0.347	1.6	1.33	1.21	2.39	1.82	1.46	1.97	2.06
Average Flexible score	0.292	0.2	1.24	1.03	1.01	1.62	1.25	1.14	1.48	1.60
Global r.m.s.d. (Å)	1.72	1.46	6.71	6.35	3.45	14.70	13.90	4.89	5.93	15.20
Best Fragment score	0.0859	0.0725	0.302	0.146	0.183	0.252	0.230	0.197	0.157	0.203
PDB code		6bbk	1qve	1oqw	1dzo	3jzz	2hi2	5v23	4d40	1oqv
UniProt ID		P18774	P17838	P02973	P02973	Q8KQ36	P02974	A0A9K2KQ72	Q8EII5	P23024

† The UniProt ID and PDB code for PilA from *P. aeruginosa* strain P1 are P17836 and 8v7p, respectively. AF ΔP1 is the *ColabFold*-generated model of ΔP1.

‡ PDB sequence alignment values are derived from the PDB files and are compared using *ProSMART*. Full-length sequence-alignment values do not consider tags or mutations in the PDB files, only the residues in the UniProt sequence starting from after the signal sequence, and are derived from *Clustal Omega*.
