# Supplementary material for: The 1.3 Å resolution structure of the truncated group Ia type IV pilin from Pseudomonas aeruginosa strain P1
Source: Acta Crystallogr D Struct Biol. 2024 Nov 28;80(Pt 12):834–49. doi: 10.1107/S205979832401132X (PMC11626772; doi:10.1107/S205979832401132X)
Supplement: Supplementary file 1 [file d-80-00834-sup1.pdf]

STRUCTURAL
BIOLOGY

Volume 80 (2024)

Supporting information for article:

The 1.3 Å resolution structure of the truncated group Ia type IV pilin from *Pseudomonas aeruginosa* strain P1

Nicholas Bragagnolo and Gerald F. Audette

S1. Details regarding the bend in the α1-C region of ΔP1

The shallow bend in the α1-C helix of ΔP1 starts at Ser41 which offsets canonical backbone hydrogen bonding of the main chain (**Fig. S1**). The carbonyl in the backbone of Ser41 H-bonds weakly with both backbone amides of Leu43 (3.08 Å) and Lys44 (3.11 Å). This conformation is stabilized by both the backbone amide and the side chain of Ser41 performing an H-bond to the carbonyl backbone of Val37 (2.78 Å). This causes a shift in the next residue, where the carbonyl group of Ala42 is bent outwards to H-bond with Gln67 of the crystallographic symmetry mate (3.11Å) rather than interacting with the backbone amide of Ala46; instead, the H-bond is bridged by water 20 (2.79 Å, 3.07 Å, 11.14 $Å^2$ B factor). Typical hydrogen bonding of the main chain resumes at Glu48 until the end of the helix at Glu53, resulting in a stretched and bent helix for one-and-a-half helical turns.

S2. Details regarding the crystallographic interface via PISA analysis of ΔP1

PISA analysis of the ΔP1 structure predicted that all potential interfaces have no role in complex formation and are a result of crystal packing only. The main crystal packing interface of ΔP1 as determined by PISA analysis consists of 77 atoms (8.4% of the protein atoms) from 29 residues (23.4% of total) of 112 surface accessible residues; this interface makes up 729 Å (10.9%) of the total solvent accessible area, 6681 Å (**Fig. S2**). The residues contributing to the highest-scoring interface include those on the solvent-exposed side of the α-helix, parts of the α-β loop and the second hypervariable loop, the first β-strand of the β-meander, and the D-loop. There are 12 hydrogen bonds occurring directly between residues of crystallographically packed neighbors, and no salt bridges or intermolecular disulfides are present for this structure, however, water molecules are seen with low B-factors serving as a bridge for the other solvent exposed residues to make hydrogen bonds that maintain the interface.

S3. Details regarding the differences in ProSMART alignments of T4P with ΔP1

The ProSMART alignment of ΔP1 with different T4Ps was performed and output aligned models were colored via the Flexible score (**Fig. 3**); when colouring residues by this method, regions can be highlighted as scoring high if they are structurally distinct as the conformation is rigid and keeps the score low if the backbone RMSD differences are merely due to dynamic regions causing conformational changes (Nicholls *et al.*, 2014). In comparing structures of ΔP1 and ΔK122 via ProSMART analysis and colouring via Flexible scoring, the 3rd β-strands of the two pilins are moderately aligned from Ile110-Arg118 in ΔP1, otherwise all regions have poor alignment (**Fig. 3*b***). Some fragments in ΔK122 are recognized as not present in ΔP1, such as regions of the ΔK122 β_1-β_2 and β_3-β_4 hypervariable loops, Ala87-Ser90 and Ala120-Asp121. These regions have residues with some of the highest B-factors in ΔP1 and are known to be important for T4P differentiation; their mobility suggests their importance in intermolecular interactions. As well, there are fragments in

ΔK122 that are not present in ΔP1; the receptor binding D-loop of ΔK122 features an extra type III β-turn from residues Lys136-Tyr137. There are also major differences in the conformation of the ΔP1 and ΔPAK pilins specifically from Ser59-Gly106 (**Fig 3*c***). Residues in ΔPAK not present in ΔP1 include Asp65-Thr67 in the α-β loop, and a residue in ΔP1 not present in ΔPAK that forms the type III β-turn, Asn85. The D-loop regions of the proteins are dissimilar, owing to a region in ΔP1 from Thr132-Trp136 not present in ΔPAK. The gap in the α1-C helix from residues Ala34-Pro42 in PAK is recognized as not being present in ΔP1 despite being recognized as highly aligned in ΔPAK, instead Ile21-Ser31 is able to conformationally align with the α1-C helix of ΔP1 (**Fig 3*c*, *d***).

In comparing ΔP1 to Δ110594, there is an additional region in the α1-C helix of the Δ110594 from Glu41-Ala53 not present in ΔP1, however the α-helix that extends to the α-β loop of Δ110594 from Ser54-Thr63 corresponds well to the α-helix of ΔP1 from Thr45-Glu53 (**Fig. 3*e***). The extended helical turn which causes the kink in ΔP1 is not well aligned from Ser41-Lys44. Regions that are not present in ΔP1 that are specific to Δ110594 include Ser69-Ser81 which constitutes the α-β loop and part of the first β-strand, and a few residues in the D-region from Thr150-Gln152.

The structure of Ng_C30 aligns with ΔP1 in a similar fashion as PAK, where the α1-N region from Glu5-Leu16 is aligned with the initial α1-C residues of ΔP1 from Ala29-Val40, however residues in the α1-C of Ng_C30 Val19-Gly42 have no counterpart in ΔP1 (**Fig. 3*f***). The next part of the α1-C from Gln43-Asn53 is partially aligned with Ser41-Glu53 of ΔP1, and the only other region of Ng_C30 with moderate structural alignment is the third β-strand from Ile101-Ala110, otherwise all regions have poor alignment to ΔP1. The α1-C of ΔNm_C8013 and ΔSo_MR-1 are highly homologous to ΔP1 up until the Ser41-Glu53 residues that cause the kink in the α-helix (**Fig. 3*g* & *h***). The third and fourth β-strands of ΔNm_C8013 align moderately well with ΔP1 from Thr116-Lys128. The regions in ΔP1 stand out as not being present in ΔNm_C8013 and ΔSo_MR-1 include large portions of the α-β loop, from Ser59-Asp65 and Gly82-Gln87 for ΔNm_C8013, from Ile57-Thr66 and Gly80-Ser84 for ΔSo_MR-1, and the D-loop region from Asn139-Ser148 for ΔNm_C8013 and the entire D-loop from Lys131-Ser148 for ΔSo_MR-1. The alignment of ΔP1 to ΔVc_RT4236 shows that only half of the α1-C region of ΔP1 aligns well with regions in the T4bP from Ser30-Gln44 and a part of the helix in the α-β loop from Ala66-Gly77; the residues in between are not considered to represent structures present in ΔP1 (**Fig. 3*i***). The rest of the T4bP is poorly aligned, with many of the hypervariable loop residues and most of the fourth β-strand not recognized in ΔP1.

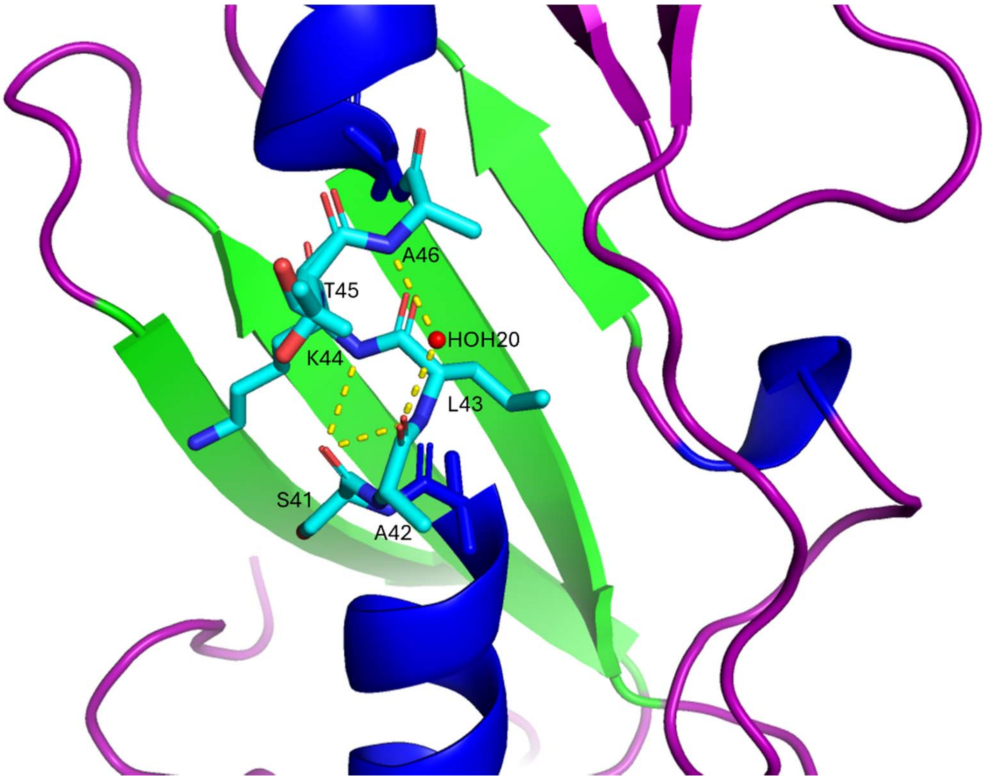

Figure S1 Relevant interactions in the hydrogen bonding network producing a bend in the α1-C region of ΔP1. The N-terminal α-helix (α1-C) is in blue with relevant residues coloured in cyan and displayed as sticks, the β-sheet is in green, the coil regions are in purple, water molecules are red balls, and hydrogen bonding is shown as yellow dotted lines. Unusual main-chain hydrogen bonding occurs between Ser41 and Leu43, Lys44 of the helix, resulting in a kinked topology. Ala42 retains a stretched helical shape through interaction with water 20 (11.14 Å^2 B factor), which H-bonds Ala46. This image was generated using PyMOL v.2.5.0 (Schrödinger, 2020).

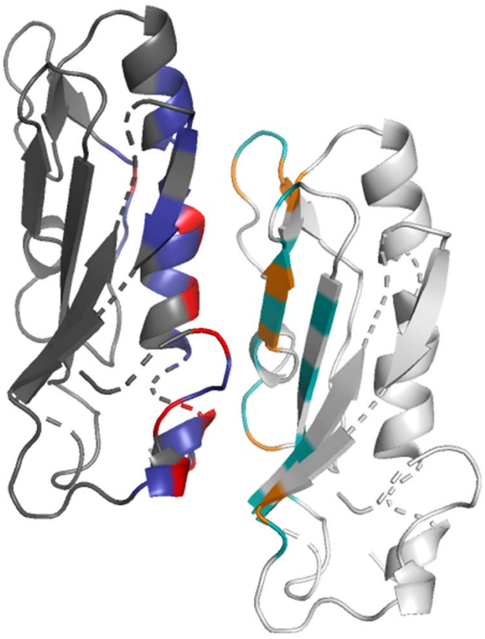

Figure S2 The crystallographic interface between two symmetry related molecules of ΔP1. The first monomer in grey shows interfacing residues in navy blue, with those making hydrogen bonds in red, while the second monomer is white with interfacing residues shown in cyan and those making hydrogen bonds in orange. Figure made in PyMOL v.2.5.0 from data obtained via PISA (Krissinel & Henrick, 2007; Schrödinger, 2020).

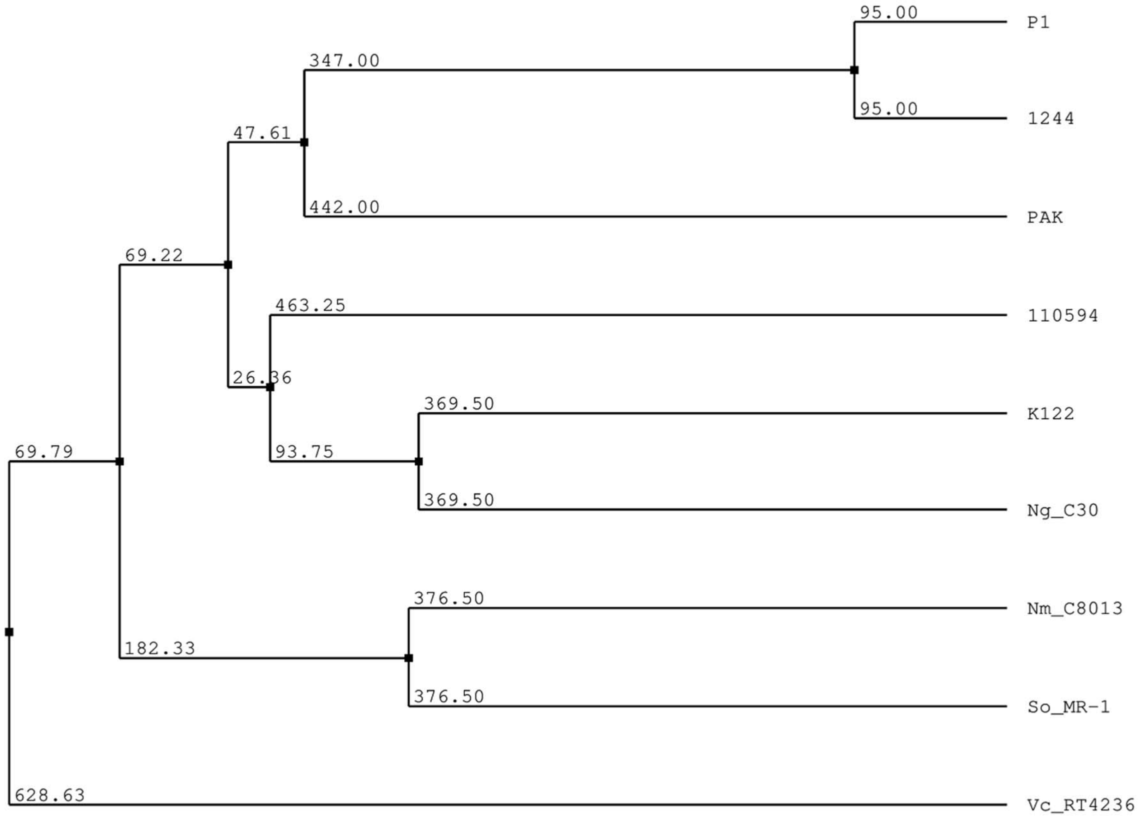

Figure S3 Phylogenetic tree displaying relatedness of T4P based on Clustal Omega alignment, scored via average distance using BLOSUM62. Image was created using Jalview (Sievers & Higgins, 2018; Thompson *et al.*, 1994; Waterhouse *et al.*, 2009).

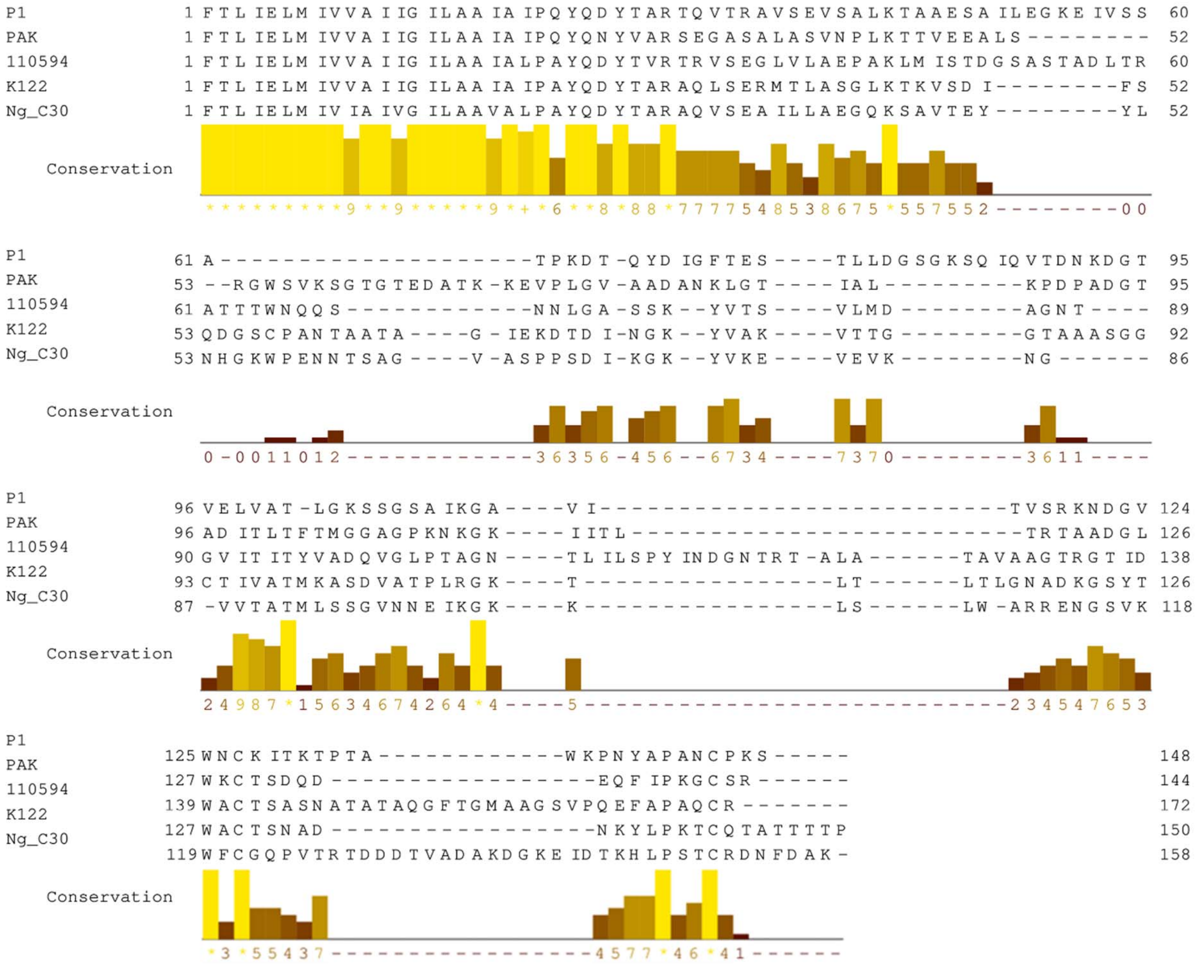

Figure S4 Clustal Omega multiple sequence alignment of T4P with solved structures similar to ΔP1. All sequences have their N-terminal signal sequence removed. Per residue conservation scores based on homology are displayed with bar graphs underneath. Ng_C30 is the major pilin PilE from *N. gonorrhoeae* strain C30. Nm_C8013, So_MR-1, and Vc_RT4236 sequences were omitted from the figure to better emphasize the sequence conservation of certain regions of the sequence-related pilins. The image was generated with Jalview (Waterhouse *et al.*, 2009).
